# Cat1 forms filament networks to degrade NAD^+^ during the type III CRISPR- Cas anti-viral response

**DOI:** 10.1126/science.adv9045

**Published:** 2025-06-12

**Authors:** Christian F. Baca, Puja Majumder, James H. Hickling, Dinshaw J. Patel, Luciano A. Marraffini

**Affiliations:** 1 Laboratory of Bacteriology, The Rockefeller University; New York, 10065, USA; 2 Tri-Institutional PhD Program in Chemical Biology, Weill Cornell Medical College, Rockefeller University and Memorial Sloan Kettering Cancer Center; New York, 10065, USA; 3 Structural Biology Program, Memorial Sloan-Kettering Cancer Center; New York, 10065, USA; 4 Howard Hughes Medical Institute, The Rockefeller University; New York, 10065, USA

## Abstract

Type III CRISPR-Cas systems defend against viral infection in prokaryotes using an RNA-guided complex that recognizes foreign transcripts and synthesizes cyclic oligo-adenylate (cOA) messengers to activate CARF immune effectors. Here we investigated a protein containing a CARF domain fused Toll/interleukin-1 receptor (TIR) domain, Cat1. We found that Cat1 provides immunity by cleaving and depleting NAD^+^ molecules from the infected host, inducing a growth arrest that prevents viral propagation. Cat1 forms dimers that stack upon each other to generate long filaments that are maintained by bound cOA ligands, with stacked TIR domains forming the NAD^+^ cleavage catalytic sites. Further, Cat1 filaments assemble into unique trigonal and pentagonal networks that enhance NAD^+^ degradation. Cat1 presents an unprecedented chemistry and higher-order protein assembly for the CRISPR-Cas response.

Clustered regularly interspaced short palindromic repeat (CRISPR) loci provide adaptive immunity to defend prokaryotes from phage (1) and plasmid (2) infection. These loci consist of DNA repeats separated by short (~30 bp) “spacer” sequences acquired from foreign, invader, genetic material (1). CRISPR arrays are transcribed and processed into CRISPR RNAs (crRNAs) that form ribonucleoprotein complexes with CRISPR-associated (Cas) proteins (3–5) and are used as guides to find invading complementary DNA or RNA sequences to start the CRISPR immune response (6–8). Type III CRISPR-Cas systems encode an effector complex with Cas10 as the main subunit, that recognizes RNA targets (7) to trigger both ssDNA degradation (9, 10) and synthesis of cyclic oligoadenylates (cOAs) from ATP substrates (11, 12). Cas10 employs its HD domain to attack ssDNA (10), an activity that can provide antiviral immunity when the target viral transcript is expressed early, but not late, in the lytic cycle (13–15). cOAs are synthesized by the Palm domain of Cas10 (11, 12), which generates 3′–5′ cyclic tri-, tetra-, or hexa-adenylate molecules that act as second messengers to activate CARF (CRISPR-associated Rossman Fold) immune effectors typically encoded in type III CRISPR loci (16). These effectors contain a CARF domain that binds cOA molecules and a second domain which activity is stimulated upon ligand binding and include RNA degradation (15, 17–19), ssDNA degradation (15, 19), dsDNA nicking (20), membrane depolarization (14) and adenosine deamination (21, 22). All of these activities are toxic to the host, and therefore CARF effectors are believed to work against phage infection through the creation of an inhospitable host that is incapable of supporting viral replication (13). As a result, CARF effectors do not rescue the infected cell but instead provide immunity at the population level. CARF effectors are essential for defense when (i) they are part of a type III CRISPR-*cas* locus harboring a *cas10* variant without an active HD domain [approximately 60 % of the known loci (23)] or (ii) when target transcript is expressed late in the phage lytic cycle and the ssDNase activity of Cas10 is unable to prevent the accumulation of viral DNA (13).

## RESULTS

### NAD^+^ degradation by Cat1 in the presence of cA4 leads to growth arrest

**Fig. 1.**
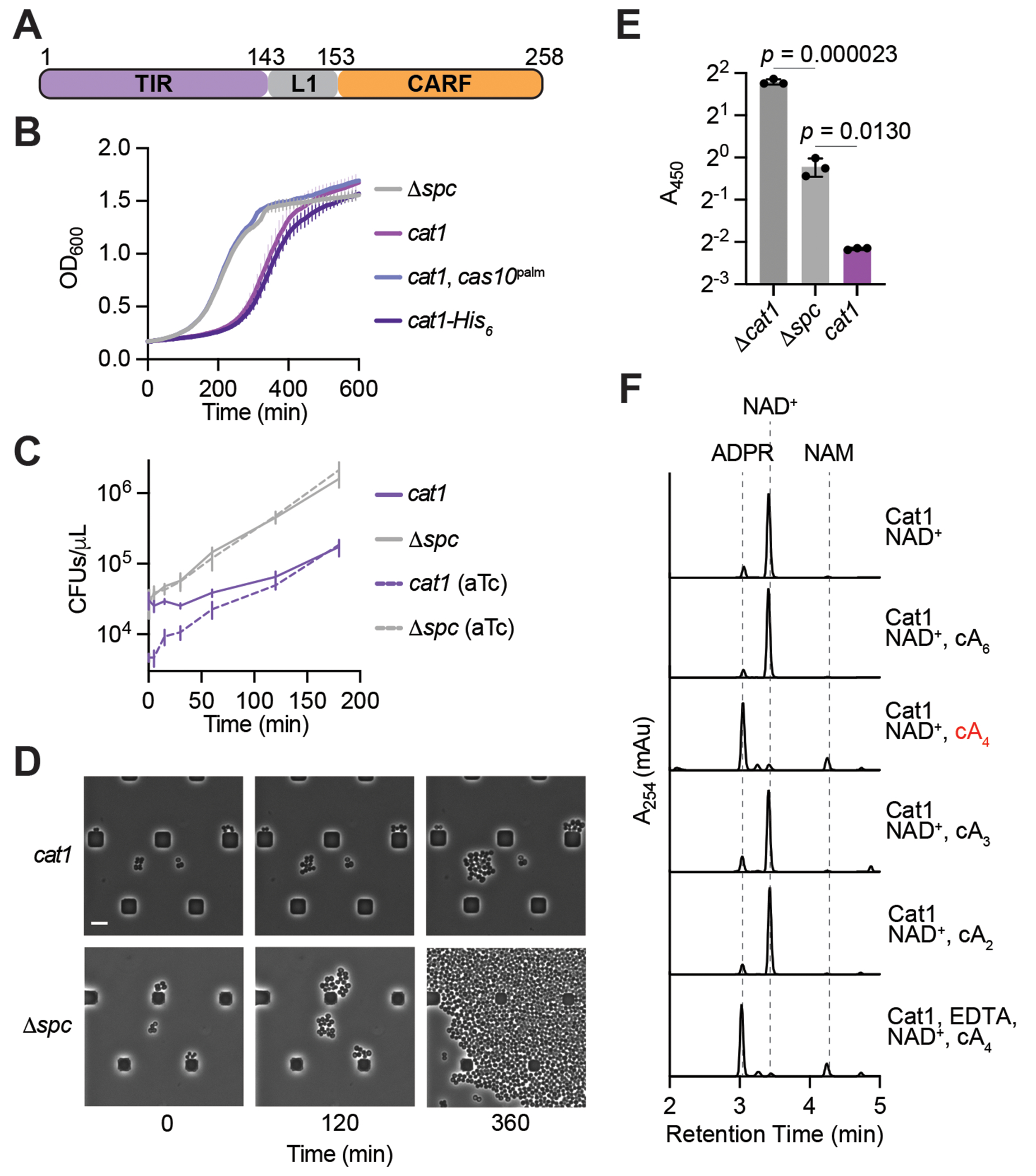
NAD^+^ depletion by Cat1 leads to growth arrest. **(A)** Domain architecture of Cat1. The protein contains a N-terminal TIR domain followed by a linker (L1), and a C-terminal CARF domain. Numbers indicate amino acids. **(B)** Growth of staphylococci carrying pTarget and different pCRISPR variants, measured as OD_600_ after the addition of aTc. Mean of three biological triplicates, ±s.e.m., is reported. **(C)** Enumeration of colony-forming units (CFU) from staphylococcal cultures carrying different pCRISPR variants after the addition of aTc. At the indicated times after induction, aliquots were removed and plated on solid medium with or without aTc to count viable cells. Mean of three biological replicates, ±s.e.m., is reported. **(D)** Time-course microscopy of *S. aureus* cells harboring pTarget and pCRISPR(D*spc*) or pCRISPR(Cat1) at different times after addition of aTc, experiment repeated for two biological replicates. Scale bar is 4 mM. **(E)** NAD(H) measurement in lysates of staphylococci harboring the indicated pCRISPR constructs and pTarget, 30 minutes after aTc addition. Absorbance at 450 nm was measured in a colorimetric assay. Three biological triplicates with ±s.e.m are reported. **(F)** HPLC chromatograms of NAD^+^ cleavage reactions. Reactions were performed with the indicated substrates at a concentration of 1 mM; Cat1, 2 mM; cOAs, 20 mM; EDTA, 1 mM; incubated at 37 °C for two hours, and then treated for HPLC separation of the substrate and the reaction products with absorbance at 254 nm as the readout.

To find novel CARF effectors, we used the CARF domain of Cam1 (14) as a query to uncover structural homologs using Foldseek (24). Our search led to the identification of a protein containing an N-terminal Toll/interleukin-1 receptor (TIR) domain fused to a C-terminal CARF domain, present in a *Chloroflexota* bacterium with accession number MBI5653133.1, which we named *Chloroflexota* CRISPR-associated TIR protein 1 (chCat1, Fig. S1A). Using PSI-BLAST we found multiple homologs of chCat1 (Fig. S1B; Data S1), many of them associated with type III CRISPR-Cas genes (Figs. S1A–B). We tested chCat1 as well as three other close homologs (Fig. S1B, accession numbers MCI0612855.1, MBW1745785.1 and NUM73787.1) for their activity in the context of the type III CRISPR-Cas response in staphylococci. We used pCRISPR, a plasmid harboring the *Staphylococcus epidermidis* RP62A type III-A CRISPR-*cas* locus (9), and replaced its CARF effector gene, *csm6* (17), by the different *cat1* homologs, and tested their ability to induce cellular toxicity, a hallmark of most CARF effectors (14, 15, 17, 21). In this assay, staphylococci carrying pCRISPR programed with a spacer to recognize a transcript produced by a second plasmid, pTarget, under the control of a tetracycline-inducible promoter (17). Addition of anhydrotetracycline (aTc) to the culture leads to the expression of the target transcript, the activation of the type III CRISPR-Cas response and the production of cOAs. We found that chCat1 and MCI0612855.1, but not MBW1745785.1 or NUM73787.1, produced a significant growth delay of staphylococci (Fig. S2A), suggesting that these Cat1 homologs are activated by cOAs. Since MCI0612855.1 displayed a more pronounced toxicity we decided to study in depth its role in the type III CRISPR-Cas immune response (this homolog is referred as Cat1 hereafter, Fig. 1A).

The ubiquitous arms race between phages and their prokaryotic hosts have resulted in the evolution of numerous antiviral defense systems besides CRISPR (25, 26), some of which employ Toll/interleukin-1 receptor (TIR) domain-containing immune effectors (26–28). Interestingly, this protein domain is utilized in immune functions throughout all domains of life, with extensive characterizations in plants (29) and mammals (30). In prokaryotes, TIR domains have been shown to play two major roles in antiphage defense. Similar to the Cas10 complex, TIR-domain proteins can recognize infection and synthesize cyclic nucleotide second messengers that activate downstream immune effectors (31–33). In addition, TIR domains have been shown to catalyze the cleavage of nicotinamide adenine dinucleotide (NAD^+^) molecules into nicotinamide (NAM) and adenosine di-phosphate ribose (ADPR), depleting infected cells of this essential metabolite to prevent phage propagation (34–36).

First, we investigated the growth arrest caused by Cat1. We found that it was abrogated in the absence of a targeting crRNA (/J*spc*) or Cas10 cyclase activity (Palm domain mutations D586A and D587A; *cas10*^palm^) (11, 12), a result that corroborates the activation of Cat1 in the context of the type III CRISPR-Cas response (Fig. 1B). To determine if the lack of growth of the culture upon activation of Cat1 is caused by cell death, we enumerated the colony forming units (CFUs) that appeared on plates lacking the inducer following plating of culture aliquots at different times after addition of aTc. We found that CFUs remained unchanged for approximately one hour (Fig. 1C), a result that demonstrates that Cat1 activation do not kill the host cells, as they are able to resume growth and form colonies when the inducer of cOA production is eliminated. After one hour, the CFU values began to rise (Fig. 1C). The same increase was observed when CFUs were counted on plates containing aTc, and therefore these colonies represent cells that are “resistant” to Cat1 toxicity, most likely due to mutations in pCRISPR or pTarget that prevent Cat1 activation (14, 17, 21). Live microscopy of cells in the aTc-induced cultures corroborated these results, revealing minimal growth of staphylococci, but no lysis, when Cat1 is stimulated by cOA production (Fig. 1D).

Given that TIR domain-containing proteins involved in anti-phage defense have been shown to degrade NAD^+^ to NAM and ADPR (31), we hypothesized that upon binding cOAs Cat1 degrades NAD^+^, thus depleting this key metabolite and causing cell toxicity. We tested this hypothesis by measuring *in vivo* levels of NAD^+^/NADH [NAD(H)] using an enzymatic colorimetric assay on lysates of different cultures treated with aTc. We found that the concentration of NAD(H) was significantly reduced when Cat1 was activated by the type III-A CRISPR-Cas response (Fig. 1E), but not in the absence of either a targeting spacer (/J*spc*) or Cat1 itself (/J*cat1*). Compared to /J*spc* cultures, staphylococci lacking Cat1 displayed significantly higher levels of NAD(H), a result that attribute to spurious production of cOAs by the Cas10 complex in the absence of a target for the crRNA guide. The growth arrest mediated by the chCat1 homolog (Fig. S2A), was also associated with a decrease in NAD(H) levels *in vivo* (Fig. S2B). We also tested Cat1 ability to cleave NAD^+^ *in vitro*. To purify the protein, we introduced a C-terminal hexahistidyl tag, generating Cat1-His6, which did not alter the toxic properties of this CARF effector (Fig. 1B). We incubated purified Cat1-His6 (Fig. S2C) with NAD^+^ and various cOA species (cA2, cA3, cA4, cA6) and analyzed the reaction products using high performance liquid chromatography (HPLC). We found that Cat1 catalyzed the cleavage of NAD^+^ into NAM and ADPR only in the presence of cA4 in a metal-independent reaction, i.e., addition of EDTA did not affect the results (Fig. 1F). Incubation of Cat1 with cA4 for an extended period of time did not change the HPLC signal of the ligand (Fig. S2D), demonstrating that Cat1 does not have detectable ring nuclease activity, as it is the case of some other CARF effectors (37). Finally, we quantified Cat1 cleavage of NAD^+^ over time using HPLC and observed a relatively linear degradation reaction (Fig. S2E). Altogether these data demonstrate that Cat1 displays NAD^+^ degradation activity that is stimulated by cA4 during the type III-A CRISPR-Cas response and that leads to the arrest of the host.

### Cat1 assembles into filaments upon cA4 binding

**Fig. 2.**
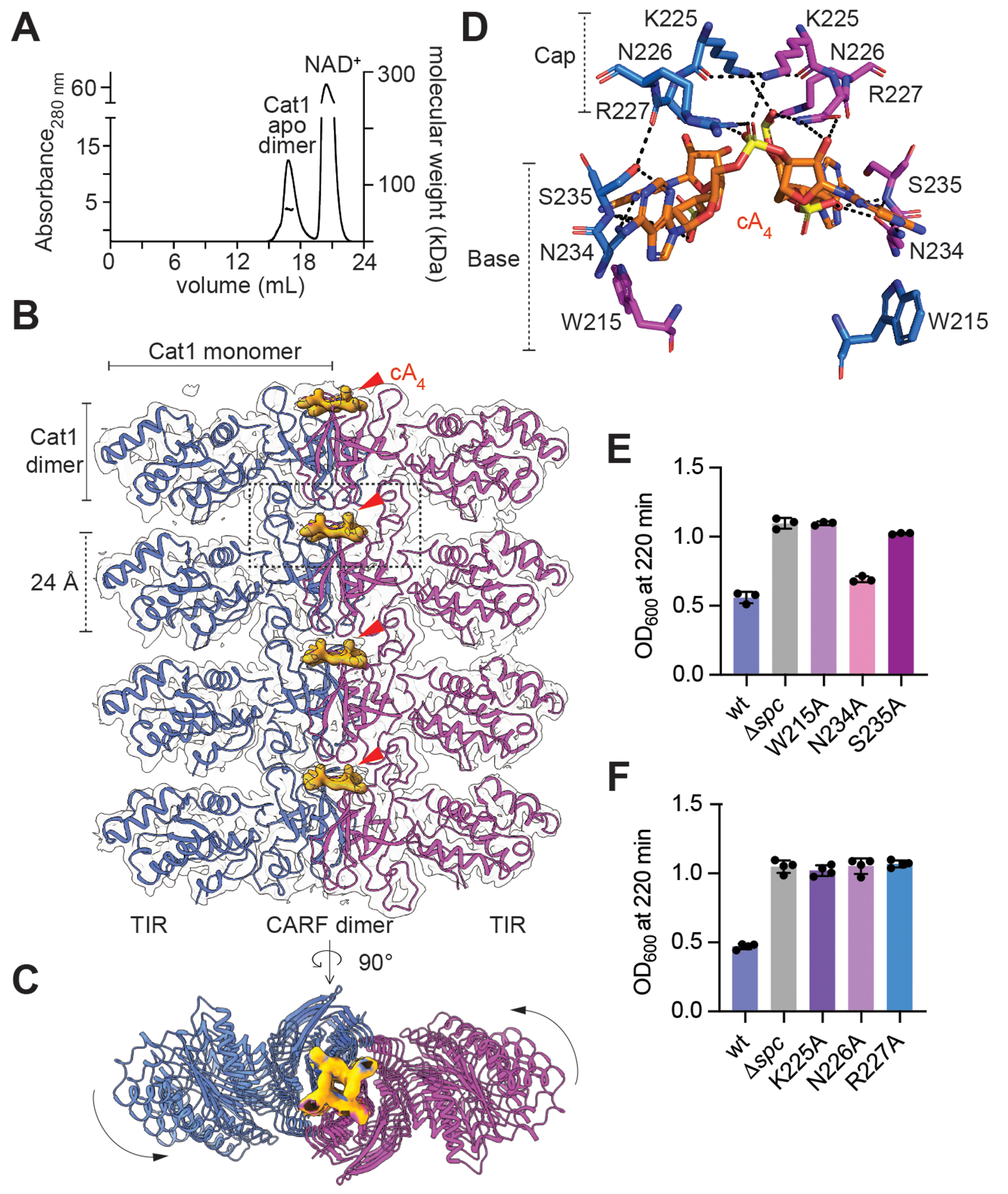
cA4 binding stimulates Cat1 filament assembly. **(A)** SEC-MALS profile of apo Cat1. The protein forms a dimer (57 kDa ± 7 %) in solution in the presence of NAD^+^. **(B)** Single filament representation of the cA4-Cat1 cryo-EM structure. Cat1 dimers form the repeat unit of the filament. Cat1 monomers are colored in blue and magenta. The center of the filament is formed by CARF dimers and TIR domains are arranged radially. The cA4 molecules “sandwiched” between the CARF dimers are presented by surface map representation in solid bright orange and indicated by red arrowheads. **(C)** Top-down view of a Cat1 filament. Black arrows show the twist of the filament assembly. cA4 density is shown bright orange. **(D)** Amino acid residues forming the base and the cap of the cA4 binding pocket formed by two CARF dimers (rectangle in panel B), represented in stick view. The residues belonging to each of the monomers are presented in blue and magenta. Black dashed lines indicate polar interactions. **(E)** Growth of staphylococci carrying pTarget and pCRISPR harboring alanine substitutions of catalytic residues in the base of the cA4 binding pocket, measured as the OD600 value after 220 minutes of addition of aTc. Mean of three biological replicates, ±s.e.m., is reported. **(F)** Same as **(E)** but with mutants in residues from the cap of the cA4 binding pocket.

Next, we performed structural studies to understand Cat1 activity at the atomic level. We first assessed the oligomeric state of the protein in the presence of its NAD^+^ substrate. In the absence of cA4 ligand, SEC-MALS analysis revealed the formation of a dimer (57 kDa ± 7 %, Fig. 2A). When incubated with the cyclic oligonucleotide, however, we noticed visible turbidity in solution, suggesting a higher order protein assembly or aggregation. We therefore prepared cryoEM grids of the cA4-Cat1-His6 complex within 10 seconds of cA4 addition and observed filament formation on the micrographs (Fig. S3A). The cryo-EM structure of cA4-bound Cat1 shows that the complex assembles into filamentous structures, forming intricate higher-order networks. The fundamental repeat unit of this topology is defined by the two-fold symmetric alignment of a pair of TIR-CARF monomers, involving central positioning of the CARF domains and outwards positioning of the TIR domains (Fig. S3B). Successive stacking of TIR-CARF dimers, anchored in place by bound cA4 positioned between them, results in formation of linear filaments (Fig. 2B), exhibiting a slight twist (Fig. 2C), as part of the higher-order filament network. CARF dimers present a two-fold symmetry (Fig. S3B) that is typical for the binding of cyclic oligoadenylates with an even number of AMP units (16). In contrast to other CARF effectors, however, the cA4 binding pocket is formed by not one but two CARF dimers, where the bottom CARF-dimer forms the base of the pocket and the top CARF dimer caps the ligand (Fig. S3C). We identified three residues from the base CARF domain dimers, W215, N234 and S235, and three from the cap CARF domains, K225, N226 and R227, as key for the interaction with cA4 (Fig. 2D).

We substituted each of these with alanine residues to test their importance for Cat1 function. We found that W215A and S235A, but not N234A (Figs. 2E, S3D), and all three cap CARF mutations, K225A, N226A, and R227A (Figs. 2F, S3E), prevented Cat1-mediated growth arrest during the type III-A CRISPR-Cas response in staphylococci. In addition, mutants containing substitutions in both cap and base residues disrupted filament formation (K225A-S235A; Figs. S3E–H) and dimerization of CARF domains (R227A-W215A; Figs. S3I–K). Together these results indicate that Cat1 filamentation is facilitated by key interactions between the cA4 ligand and CARF dimers on either side of a growing filament. Cat1 therefore presents a unique structure for a CARF effector in which cA4 molecules that are sandwiched between CARF domains act as a glue for the assembly of a long filament.

### Higher-order filament networks enhance Cat1 NAD^+^ degradation activity

**Fig. 3.**
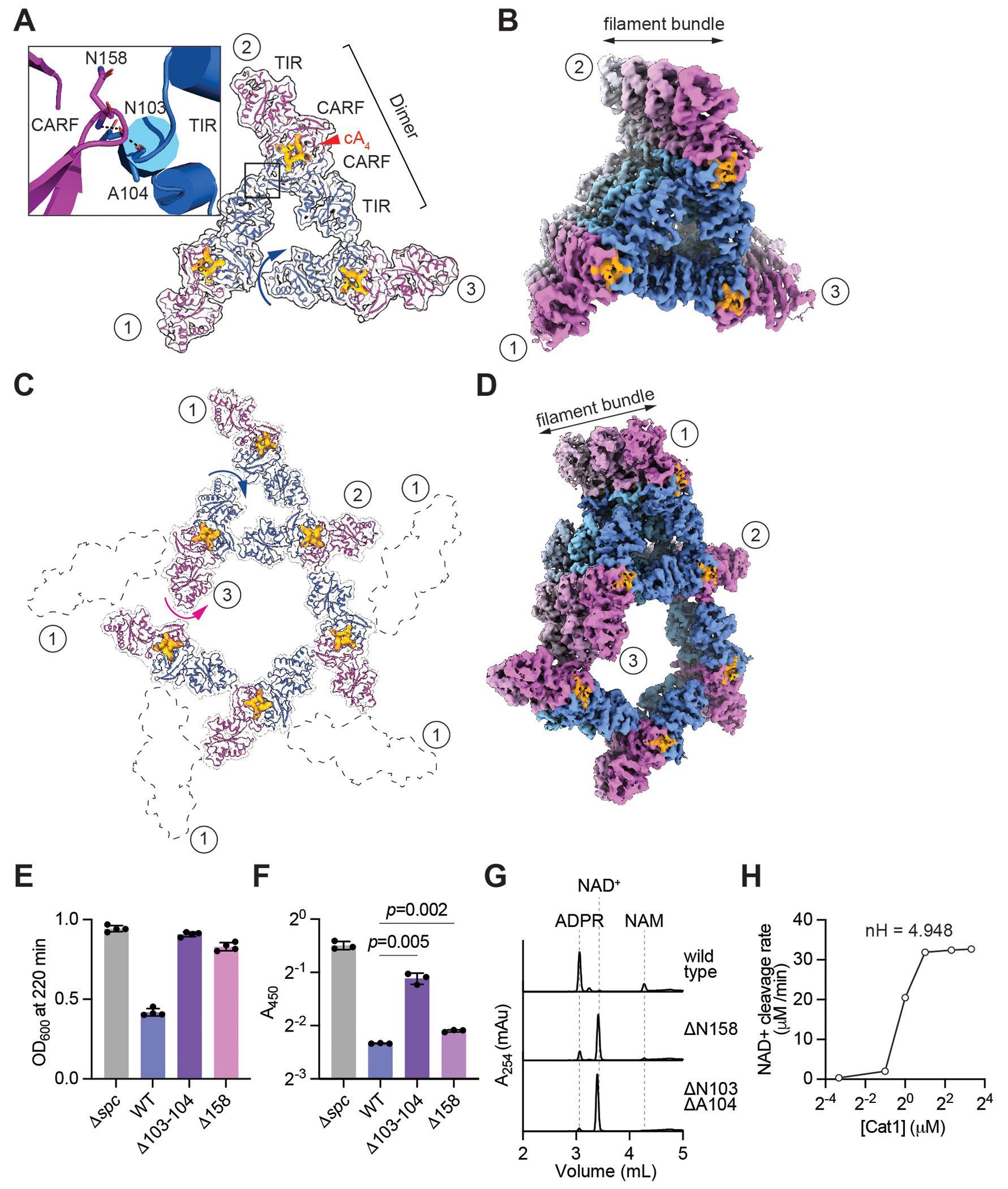
Higher-order filament networks enhance NAD^+^ degradation. **(A)** Trigonal filament assembly of the cA4-Cat1 complex formed by inter-repeat unit interactions indicated by the black rectangle. Circled numbers indicate each of the three filaments. Inset: magnified view of the inter-repeat unit interaction showing key TIR and CARF residues. cA4 density is shown in bright orange and indicated by a red arrowhead. Cat1 dimer “3” is tilted around its center which shifts the TIR domain belonging to the blue Cat1 monomer, below the plane of Cat1 dimers “1” and “2”, indicated by the blue arrow. **(B)** Surface representation of the cryo-EM map of the trigonal helical filament bundle of cA4-Cat1 complex. Each turn of the bundle has three Cat1 dimers labelled as circled numbers. The successive turns are arranged without any gap (zero pitch) between them and shown by the gradient of lighter shades of magenta and blue for Cat1 monomers to designate different turns. The double-headed arrow indicates the elongation of the trigonal filament bundle. **(C)** Cryo-EM structure of the pentameric cA4-Cat1 complex. The tilt of the third Cat1 dimer is shown by blue and magenta arrows to indicate shifts of the TIR domains above and below the plane, respectively. The segment of the assembly with broken densities are marked as “1” and are shown by a dashed black border. Model building was not performed for these segments. **(D)** The surface representation of the pentameric filament bundle of the cryo-EM map of cA4-Cat1 complex is shown. The double-headed arrow displays elongation of the helical (zero pitch) filament bundle and the successive turns are displayed with the same color scheme as **(B)**. **(E)** Growth of staphylococci carrying pTarget and pCRISPR harboring amino acid deletions of the residues involved in filament-filament interactions, measured as the OD600 value after 220 minutes of addition of aTc. Mean of four biological replicates, ±s.e.m., is reported. **(F)** NAD(H) measurement in lysates of staphylococci expressing Cat1(/J158), 30 minutes after aTc addition. Absorbance at 450 nm was measured in a colorimetric assay. Four biological replicates with ±s.e.m are reported. **(G)** HPLC chromatograms of NAD^+^ cleavage reactions. Reactions were performed with the indicated substrates at a concentration of 1 mM. The indicated Cat1 mutants were used at a concentration of 1 mM; cOAs, 10 mM. Reactions were incubated at 37 °C for two hours, then treated for HPLC separation of the substrate and the reaction products with absorbance at 254 nm as the readout. **(H)** NAD^+^ cleavage rates plotted as a function of Cat1 concentration. Values were calculated with the data collected in Figure S4I. Hill coefficient (nH) was calculated from a sigmoidal non-linear regression fit.

While analyzing the higher-order filament network of cA4-Cat1 complex, we noticed that the dimeric repeat units associate with two adjacent Cat1 dimers forming a surprising trigonal assembly (Fig. 3A, Fig. S4A). The inter-repeat unit association is mediated by amino acid backbone interactions between a small loop formed by N158 and G159 residues joining the β1- β2 strands of the CARF domain (designated β1β2-loop) of repeat unit 1 and the N103 and A104 residues present at the TIR domain of the adjacent Cat1 dimer (Fig. 3A, Fig. S4B). In this trigonal assembly, the third Cat1 dimer does not pair up with the first Cat1 dimer, but instead it tilts around the center of the dimer (see blue arrow implying a shift below the plane, Fig. 3A). The tilt causes a shift of the TIR domain, one of which points towards the trigonal assembly, below the plane of repeat units 1 and 2 by 24 Å, thereby forming the helical bundle by bringing it close to the CARF domain β1β2-loop of the Cat1 dimeric unit which initiates the next turn (Fig. 3A). Thus, the trigonal Cat1 dimer assembly gives rise to a spiral filament bundle with a triangular pore in the middle (Fig. 3B). Three Cat1 dimers are present in each turn of the spiral bundle with an overall diameter of 170 Å and the bundle is closely packed without any pitch (gap between turns) (Fig. 3B).

To our surprise, we observed that the trigonal filament assembly further associates with more Cat1 filaments generating a pentameric assembly (Fig. S4C). The inter-repeat unit interaction between β1β2- loop on both sides of a dimeric CARF domain of a Cat1 dimer and the TIR domain of the adjacent Cat1 dimer, enables the assembly extension and provides an opportunity to form an even larger filament network (Fig. 3C). In the cryo-EM 3D classification, we observed two distinct classes, one corresponds to the trigonal filament bundle, described previously, and the other where five trigonal filament bundles join to form a large pentameric assembly (Fig. 3C). As the filament network spreads, flexibility results in broken densities (marked as “1” in black dashed lines, Fig. 3C), preventing model building for these segments. The tilt of the third Cat1 dimer around the center of the repeat unit of the trigonal filament bundle shifts the TIR domain, which points opposite to the trigonal filament assembly, above the plane with respect to the of rest of the Cat1 dimeric units of the pentameric assembly (see magenta arrow implying shift above the plane; Fig. 3C). The above-mentioned shift of the TIR domain mediates its interaction with the Cat1 dimer of the next turn and results in the generation of a closely packed spiral bundle without pitch and with a large pentagonal pore at the center (Fig. 3D).

To test the importance of these supra-structures for Cat1 activity, we made deletions of residues N103-A104 and N158 to eliminate the backbone interactions that facilitate high-order assembly (Fig. 3A, inset; Fig. S4B). The hexahistidyl-tagged versions of these mutants were purified and subjected to size exclusion chromatography, which showed that the amino acid deletions did not prevent the folding of Cat1 (Figs. S4D, S4E). Cryo-EM analysis of the purified proteins in the presence of cA4 demonstrated the disruption of higher order filament assembly for both mutants at 165,000 magnification, with only small filament fragments remaining (Figs. S4F, S4G). This result further supports the idea that the linear (Fig. 2B), trigonal (Fig. 3B) and pentagonal (Fig. 3D) filament bundles of Cat1 are not independent units, but part of the same filament network, and hence cannot be investigated on their own. *In vivo*, upon activation of the type III-A CRISPR-Cas response, both deletion mutants lost their toxicity (Figs. 3E and S4H). While staphylococci expressing Cat1(/JN158) displayed a small but yet significant increase in NAD(H) levels compared to cells harboring wild-type Cat1 (a consequence of a much slower NAD^+^ cleavage reaction, as measured *in vitro* using different substrate concentrations; Fig. S4I), activation of Cat1(/JN103-A104) failed to reduce the cellular concentration of this metabolite, similarly to a non-targeting control (/J*spc*) (Fig. 3F). *In vitro* reactions revealed that both mutations notably reduced NAD^+^ cleavage by Cat1 (Fig. 3G). Finally, we evaluated the kinetics of filamentation and disassembly. First, we measured substrate and product levels after an incubation period of 30 minutes with different Cat1 concentrations (Fig. S4J). We calculated the average NAD^+^ degradation rates and observed a sigmoidal increase with Cat1 concentration (Fig. 3H), with a Hill coefficient of 4.954 that indicates a strong positive cooperativity. Given the importance of filamentation for NAD^+^ degradation, we conclude that a critical concentration of Cat1 is required for the formation of high-order structures and thus the achievement of maximum NAD^+^ degradation within the host cell. Second, we investigated filament disassembly *in vivo*, by providing a pulse of cA4 production treating a culture harboring pTarget with aTc and quantifying intracellular levels of NAD^+^. We carried out measurements before and 30 minutes after aTc addition and, consistent with the results in Figure 1E, we detected much lower levels of NAD^+^ than in a non-targeting control culture (/J*spc*, Fig. S4K). We then washed staphylococci to eliminate the aTc inducer and stop cA4 production and continue measuring NAD^+^. For the first two hours after de-activation of Cat1, the intracellular NAD^+^ concentration remained low and then increased, presumably due to the cessation of NAD^+^ cleavage upon disassembly of Cat1 filaments, reaching the same levels detected in the control culture. Together, these results show that the formation of filament networks enhances Cat1’s catalytic activity, most likely through the increase of the amount of catalytically competent protein or through the stabilization of the catalytically competent structure. These networks, however, are not stable and can disassemble over time.

### Filamentation is required for the formation of the Cat1 active site

**Fig. 4.**
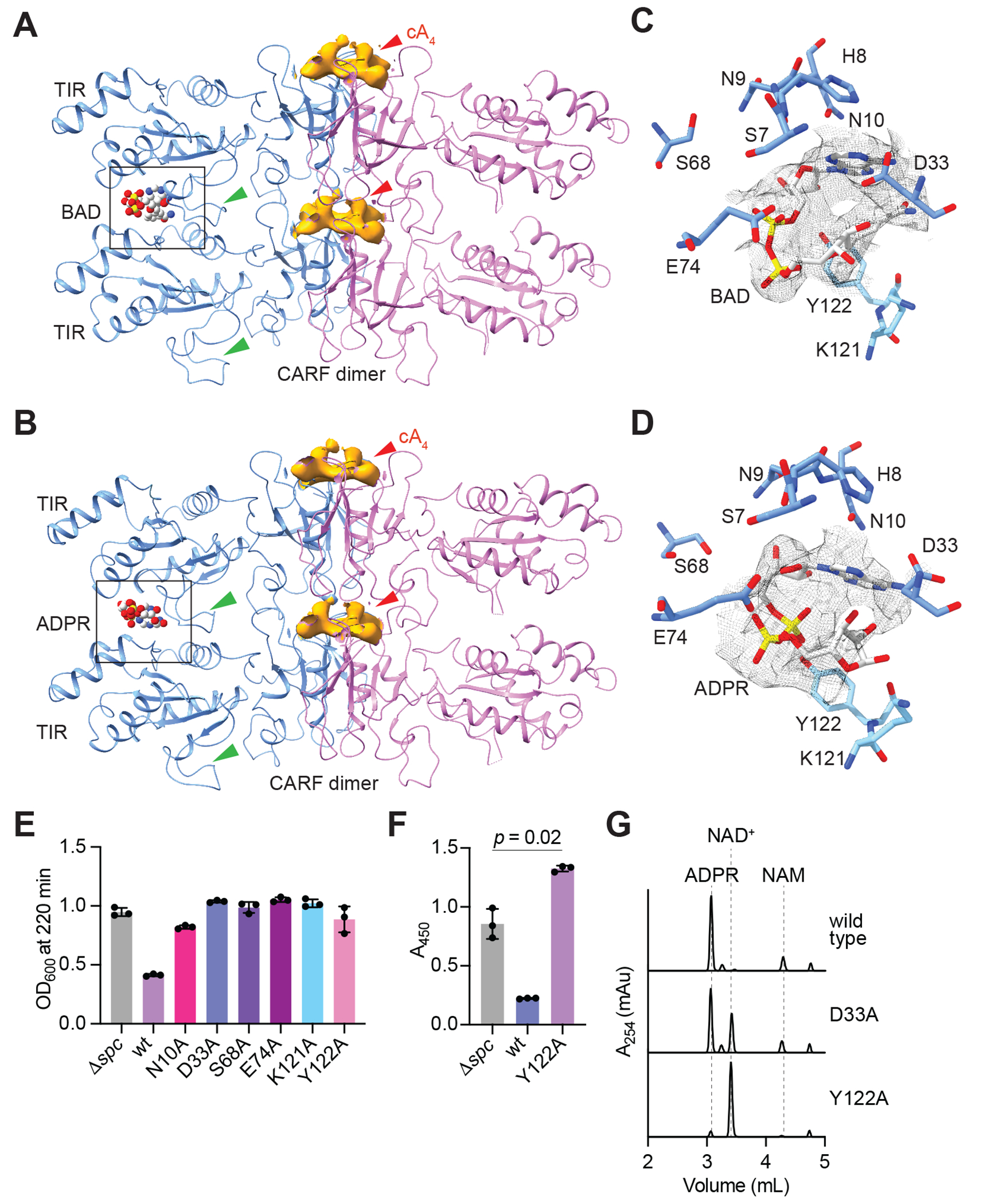
Filamentation is required for the formation of Cat1 active site. **(A)** Cryo-EM structure of cA4-BAD-Cat1-His6 showing a pair of Cat1 dimers with modelled BAD at the NADase active site (black rectangle) formed by two TIR domains. cA4 density is shown bright orange and indicated by a red arrowhead. The BB-loop of the TIR domain is shown by a green arrowhead. **(B)** Cryo-EM structure cA4-NAD^+^-Cat1-His6 showing the same view as in **(A)** with modelled ADPR at the NADase active site. **(C)** NADase active site with modelled BAD in the cA4-BAD- Cat1-His6 structure. Residues are shown in stick representation. The residues from different TIR domains are designated in dark and light blue. The coulomb potential map for the modelled BAD molecule is shown in grey (contour level 2.9σ). **(D)** Same as **(C)** for modelled ADPR (contour level 4σ) in the cA4-NAD^+^-Cat1-His6 structure. **(E)** Growth of staphylococci carrying pTarget and pCRISPR harboring alanine substitutions of catalytic residues in the NADase active site, measured as the OD600 value after 220 minutes of addition of aTc. Mean of three biological replicates, ±s.e.m., is reported. **(F)** NAD(H) measurement in lysates of staphylococci expressing Cat1 with alanine substitutions in key residues of the NADase active site, 30 minutes after aTc addition. Absorbance at 450 nm was measured in a colorimetric assay. Three biological replicates with ±s.e.m are reported. **(G)** HPLC chromatograms of NAD^+^ cleavage reactions. Reactions were performed with the indicated substrates at a concentration of 1 mM. The indicated Cat1 mutants were used at a concentration of 1 mM; cOAs, 10 mM. Reactions were incubated at 37 °C for two hours, then treated for HPLC separation of the substrate and the reaction products with absorbance at 254 nm as the readout.

To understand the effects of Cat1 filamentation on the catalysis of NAD^+^ cleavage at the atomic level, we solved cryo-EM structures of cA4-bound Cat1 in the presence of a non-hydrolysable NAD^+^ analogue, benzamide adenine dinucleotide (BAD) or NAD^+^ at 3.3 Å and 4 Å resolution, respectively. We could resolve similar pentameric filament network formation for class 1 that represented the largest number of particles for both cA4-BAD-Cat1-His6 (Fig. S5A and S10B, red box) and cA4-NAD^+^-Cat1-His6 (Fig. S5B and S11B, red box) and therefore these were processed further, to the exclusion of other classes in both complexes that were part of the same network. We modelled a pair of Cat1 dimers in the map for each structure (Figs. 4A, 4B). In the cryo-EM maps for both complexes the center of the filament was very well resolved whereas the loops of the radially arranged TIR domains displayed poor quality. Therefore, we built models for those parts of the map where we could partially or fully model the BB-loop of the TIR domain (green arrowhead in Figs. 4A, 4B). The BB-loop is predicted to control the access to the NADase active site in other TIR proteins (38–40). Both structures revealed that the active site is formed by two TIR domains from a pair of stacked Cat1 dimeric repeat units, suggesting that the catalytic activity relies on the multimerization of Cat1 dimers upon cA4 signal recognition (Figs. 4A, 4B). We were able to model the BAD molecule in the modestly resolved density obtained for the NADase pocket for cA4-BAD-Cat1-His6 structure (Fig. 4C). In the case of the cA4− NAD^+^-Cat1-His6 structure, the observed density was sufficient to model ADPR, but neither the NAD^+^ substrate nor the other reaction product, NAM (Fig. 4D). In both cases we observed that residues S7, H8, N9, N10, D33, S68 and E74 from one TIR domain, and K121 and Y122 from the other, line the NADase active site (Figs. 4C, 4D). We tested the importance of these residues for Cat1 activity by making alanine substitutions (N10A, D33A, S68A, E74A, K121A and Y122A). The mutants were tested *in vivo* by measuring their toxicity during the type III-A CRISPR-Cas response and were all found to be defective in causing a growth arrest in staphylococci (Figs. 4E and S5C). Consistent with this result, staphylococci expressing Cat1(Y122A) displayed high levels of NAD(H) (Fig. 4F); similarly higher than the /J*spc* control as in cells that do not express Cat1 (Fig. 1E). We also purified the D33A and Y122A Cat1-His6 mutants (Figs. S5D, S5E) to analyze the impact of these catalytic residues in NAD^+^ cleavage reaction *in vitro*. While the D33A substitution reduced activity only partially, the Y122A mutation completely abrogated NAD^+^ cleavage (Fig. 4G). Interestingly, the complete absence of toxicity for the D33A mutant *in vivo* suggests that staphylococci can adapt to low levels of NAD^+^ depletion. Altogether these data showed that Cat1 active site is formed through the interaction of two stacked TIR domains from different dimers, a finding that provides an explanation for the importance of filamentation for its enzymatic activity, as well as for the cooperativity of NAD^+^ cleavage (Figs. 3E–H).

### Cat1 provides anti-phage defense

**Fig. 5.**
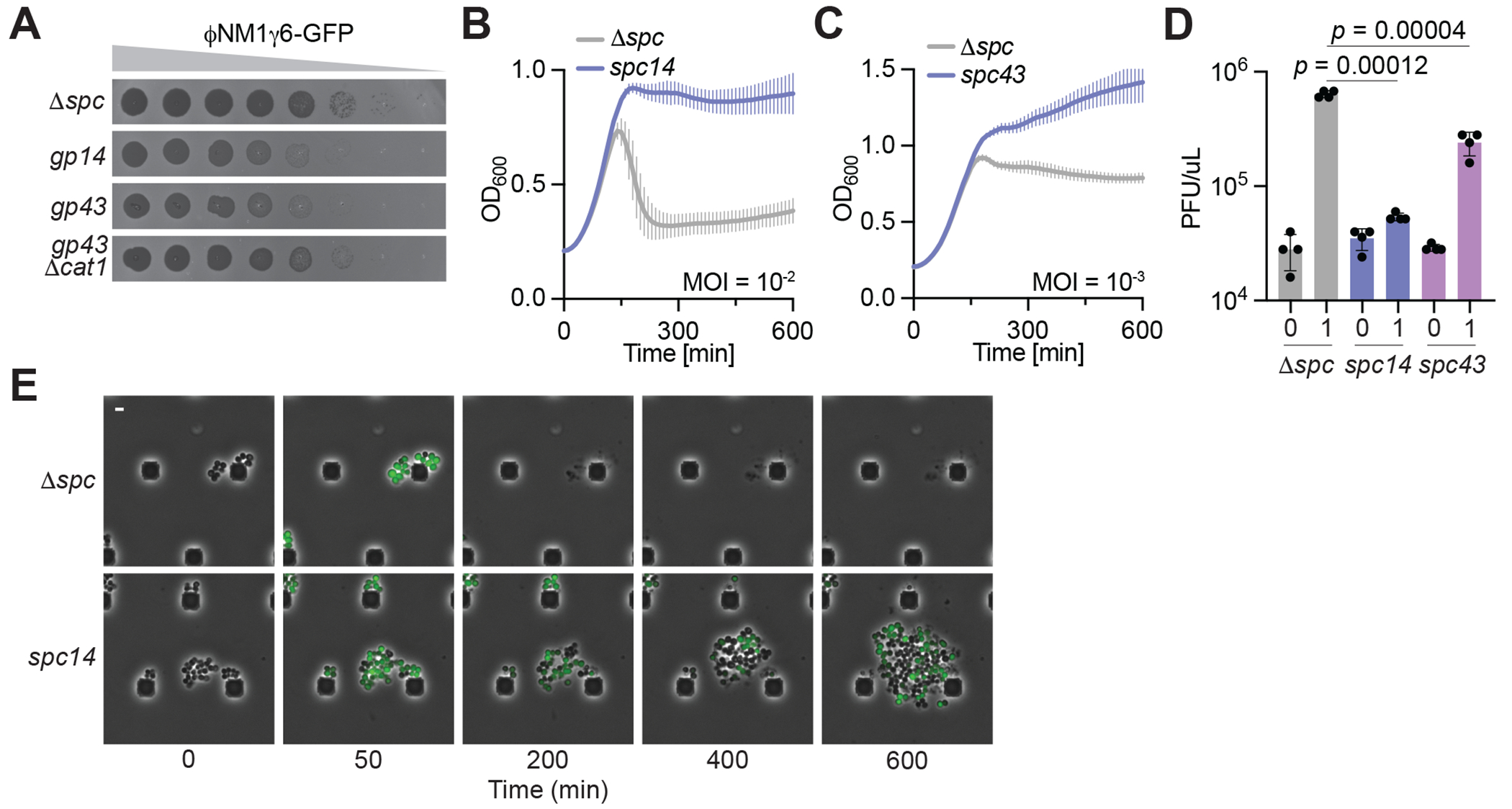
Cat1 mediates anti-phage immunity. **(A)** Plaquing of ΦNM1γ6-GFP on *S. aureus* lawns harboring pCRISPR(*cas10*^HD^) programmed with spacers to target an early- (*gp14*) or a late- expressed (*gp43*) viral gene, in the presence or absence of Cat1 (/J*cat1*), or a non-targeting control (/J*spc*). Images are representative of one of three biological triplicates. **(B)** Growth of staphylococci carrying different pCRISPR constructs programmed with *spc14* or a non-targeting spacer (/J*spc*), measured as OD600 of the cultures after infection at the indicated MOI. Mean of three biological triplicates, ±s.e.m, is reported. **(C)** Same as in **(B)** but with a pCRISPR construct programmed with *spc43*. **(D)** Enumeration of plaque-forming units within staphylococcal cultures harboring different pCRISPR constructs programmed to target ΦNM1γ6-GFP with the indicated spacers, either before (“0”) after one hour (“1”) of infection with ΦNM1γ6-GFP at an MOI of ∼1. Mean of three biological replicates, ±s.e.m., is reported. *p* values, obtained with a two-sided t test with Welch’s correction, are shown. **(E)** Time course fluorescence microscopy of *S. aureus* harboring different pCRISPR constructs after infection with ΦNM1γ6-GFP. Images are representative of two biological replicates. Scale bar, 4 μM.

For the majority of CARF effectors studied to date, the toxicity they mediate is essential to provide anti-phage immunity in the absence of Cas10’s nuclease activity (13–15, 21, 41). Therefore, to test the function of Cat1 in the type III-A CRISPR-Cas response against phage, we mutated *cas10* in pCRISPR to change amino acid residues of the nuclease active site that are key for ssDNA degradation (H14A, D15A; *cas10*^HD^) (17), and introduced spacers that target transcripts from different phages (Fig. S6A). For the phage fNM1g6-GFP (14), spacers that target both early- and a late-expressed transcripts (*spc14* and *spc43*, respectively) mediated a reduction in the plaque forming units (PFU) when ten-fold dilutions of the phage stock were plated on lawns of staphylococci carrying the different pCRISPR plasmids (Fig. 5A). As expected, immunity was abrogated when we infected lawns of bacteria carrying a *cat1* deletion in the pCRISPR plasmid (/J*cat1*; Fig. 5A). Both spacers also prevented the collapse of liquid cultures after addition of phage at low multiplicity of infection (MOI), although immunity for spc14 (Fig. 5B) was attained at an MOI 10 times higher than for spc43 (Fig. 5C). This result was corroborated after enumerating the PFU present in the infected cultures one hour after infection, plating on lawns of bacteria that lack any phage defense (Fig. 5D). To investigate these results at the cellular level, we performed fluorescence microscopy, taking advantage of GFP expression by fNM1g6-GFP to visualize infected cells (13, 14). Consistent with the toxicity mediated by Cat1 activation that we observed in the presence of pTarget (Figs. 1B–D), the addition of phage to cultures carrying pCRISPR(*spc14*) led to a growth arrest of the infected cells (marked by green fluorescence) that was followed by the division of non-infected ones (non-fluorescent) (Fig. 5E). In addition, we also observed lysis of some of the infected staphylococci. This is in line with other reports of CARF effectors that provide immunity to the population by preventing the spread of the virus and therefore promoting the replication of uninfected bacteria (13, 14, 21). Similar to the previous results for *spc43*, we found that most cells treated with fNM1g6-GFP succumbed to infection and lysed (data not shown). Therefore, at least upon heterologous expression in staphylococci, Cat1 provides stronger immunity when it is activated early in the type III-A CRISPR-Cas response, i.e., when the RNA-guided Cas10 complex initiates cA4 synthesis after recognition of an early-expressed transcript. Finally, we tested type III-A CRISPR-Cas immunity against diverse staphylococcal phages (42) using strains carrying pCRISPR(*cas10*^HD^) plasmids programmed with different targeting spacers (Fig. S6A). We found that Cat1 both reduced viral propagation of approximately two orders of magnitude for phages f12g3 (43), fNM4g4 (44), f80a-*vir* (42), fJ1 (42) and fJ4 (Figs. S6B–F) (42), as well as supported the growth of staphylococci infected with f12g3 and f80a-*vir* (Figs. S6G–I).

## DISCUSSION

**Fig. 6.**
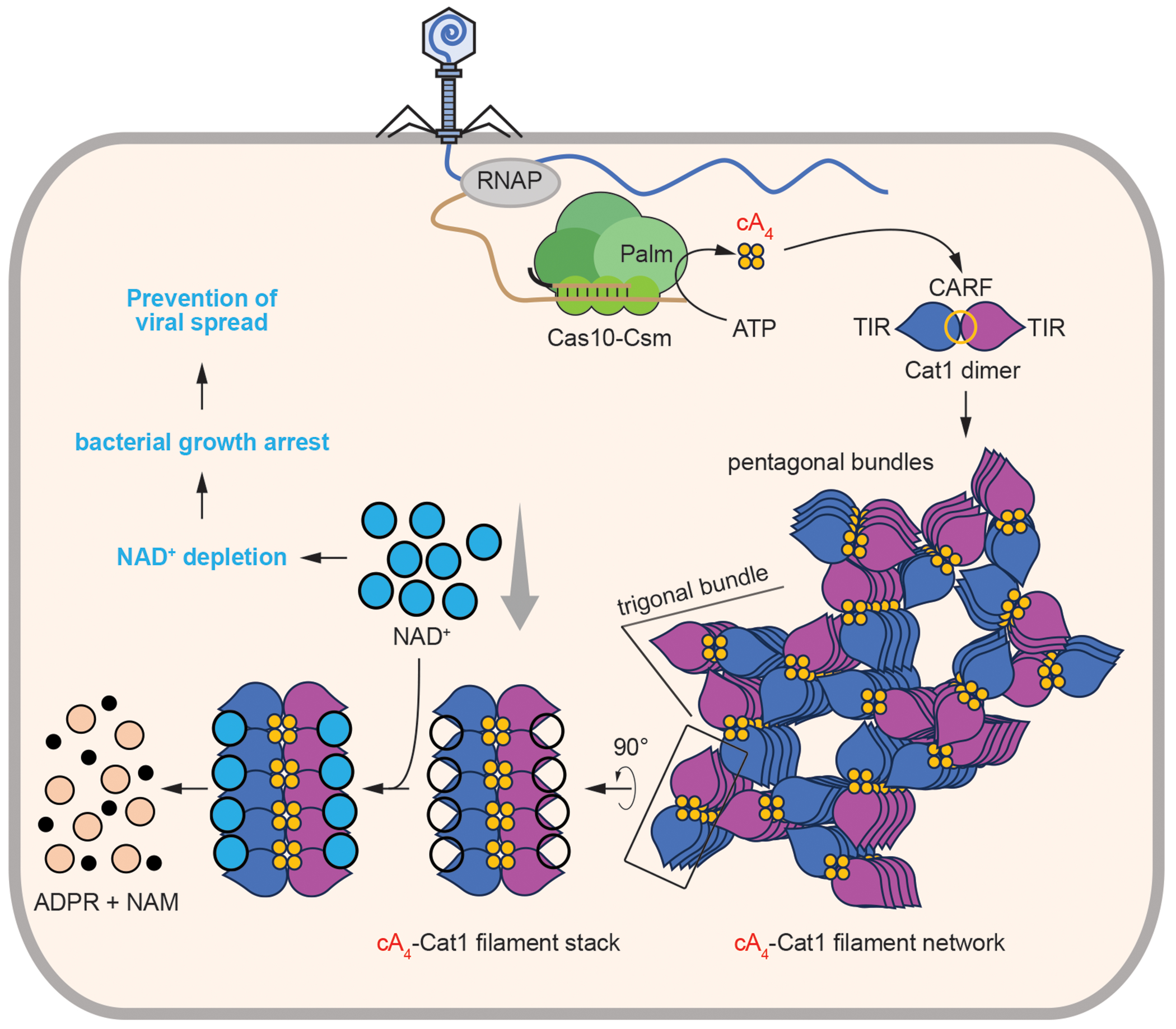
Cat1 filament network provides phage immunity by NAD depletion. Upon detection of viral transcript by type III CRISPR-Cas complex guided by CRISPR-RNA, Cas10 synthesizes cyclic tetra-adenylates (cA4) messenger molecules that bind to the Cat1 dimer to trigger dimer stacking and the formation of long filaments. These filaments further expand into a filament network formed by trigonal and pentagonal filament bundles. The stacked TIR domains of this filament assembly form the NAD^+^ binding pocket which catalyzes the cleavage of the substrate into adenosine diphosphate ribose (ADPR) and nicotinamide (NAM). Depletion of cellular NAD^+^ causes a bacterial growth arrest and prevents viral spread to the rest of the bacterial population.

Here we describe the function and structure of Cat1, a type III-associated CARF effector. Upon binding cyclic oligoadenylates synthesized when the RNA-guided Cas10 complex finds a viral target transcript, Cat1 forms long filaments that assemble into higher order structures. Filamentation creates a binding pocket for Cat1’s substrate, NAD^+^, and filament assembly stimulates NAD^+^ degradation. Therefore, Cat1 activation leads to NAD^+^ depletion and promotes an arrested state of the host cell that cannot support viral replication (Fig. 6). Given the growth arrest of the infected cells, uninfected cells that are able to continue growing make the bulk of the surviving population. We found that in the absence of ssDNA degradation by the type III Cas10 nuclease, Cat1 provides better immunity when it is activated early during infection, a result that suggests that the depletion of NAD^+^ mediated by Cat1 cannot effectively stall phage propagation late in the lytic cycle, when most viral DNA replication has already happened. This is also the case for other CARF effectors that have been studied through heterologous expression in staphylococci, such as Card1 (15), Cam1 (14) and Cad1 (21). In contrast, in the absence of Cas10 ssDNase activity, the native staphylococcal CARF effector Csm6 provides strong population immunity (13), and therefore we speculate that Cat1 could also mediate strong defense in a *cas10*^HD^ background in its native hosts. This is important because approximately only 40% of type III CRISPR-Cas systems contain variants that either lack an HD domain (23) or do not display detectable ssDNase activity (45). In the case of *cat1*, only ~10 % of the homologs were found associated with a *cas10* gene displaying an intact HD domain (Data S1).

Cat1 activation in the absence of phage showed that staphylococci pause their growth but do not die, as they are able to form colonies after the synthesis of cA4 is stopped (Fig. 1C). Live microscopy of cultures where Cat1 was activated either via induction of a plasmid-based target transcript (Fig. 1D) or after phage infection (Fig. 5E) did not reveal significant cell lysis. Absence of cell death, as well as the increase of intracellular NAD^+^ levels upon Cat1 deactivation *in vivo* (Fig. S4J), suggest that Cat1 activation does not lead to a rapid and complete NAD^+^ depletion and raises questions about potential mechanisms for the disassembly of Cat1 networks, which, given that higher-order filament assembly is required for maximum catalysis, would significantly reduce the rate of NAD^+^ degradation. One possibility to transiently deactivate Cat1 would be the cleavage of the cA4 ligand, a property of some CARF effectors that leads to on-off cycles that generate “bursts” of toxic activity (37). However, we did not detect such cleavage (Fig. S2D). We anticipate a lateral spreading of the filament network of Cat1, with poor density of the filaments as they spread (Fig. S7A), suggesting that the inter-filament interactions that hold larger assemblies together are weak. It is currently difficult to speculate any mechanism of filament disassembly based on the structural data. Another possibility for the lack of cell death upon Cat1 activation is the relatively low rate of NAD^+^ degradation that was observed (Fig. S2E). Growth arrest without cell death was also observed for other CARF effectors (13, 14, 17, 21), but does not seem to be the case during the Thoeris anti-phage defense (31), a prokaryotic immune system in which the SIR2 domain-containing effector, ThsA, also forms long filaments that degrade NAD^+^ (46).

We compared the cryo-EM structure of Cat1 filament with the model predicted by AlphaFold3 (AF3) (47) and we found a completely different assembly where TIR domains form the center of the filament and CARF monomers are radially arranged (Fig. S7B). This is due to a failure to predict the dimerization of CARF domains, a hallmark of all CARF effectors. On the other hand, AF3 provided a correct guess for the architecture of the NADase pocket (Fig. S7B, black box). This result suggests that the head-to-tail arrangement of TIR domains is well conserved evolutionarily, and thus over-represented in the protein structure database; including TIR-domain containing proteins of higher eukaryotes such as human SARM1 (48). Structurally, Cat1 is unique among anti-phage effectors that assemble into filaments to provide defense. For example, ThsA, the effector of the Thoeris system, assembles into a helical filament upon binding its activating ligand, 1’’−3’ glycocyclic ADP ribose (gcADPR) (46). In contrast to Cat1, where the CARF dimers form the central part of the filament, the ThsA filament is formed by a tetrameric SIR2 domain (Fig. S7C). Also unlike Cat1, the Lon-SAVED (49) and TIR-SAVED (34) fusion proteins associated with CRISPR and cyclic oligonucleotide-based anti-phage signaling systems (CBASS), as well as the TIR-STING effector associated with CBASS systems (50), these effectors share a filament architecture in which the cyclic nucleotide-binding domains (SAVED and STING) are arranged on one side of the filament and catalytic domains (Lon protease and TIR) are displayed on the other side of the filament (Figs. S7D–F). In addition to the distinctive arrangement of sensor and catalytic domains, the higher-order networks formed by inter-filament association are unique to Cat1. Finally, Cat1 enzymatic activity is also unprecedented among CRISPR-Cas systems. To date CARF effectors have been shown to provide immunity via nucleic acid cleavage (15, 17–20), membrane depolarization (14) and ATP deamination (21, 22). Our findings for Cat1 add NAD^+^ cleavage to this list, and therefore reveal a wide-range of mechanistic flexibility for the CRISPR-Cas defense.

## Materials and Methods

### Phylogenetic analysis of Cat1

Cat1 homologs were identified with a PSI-Blast search against the original Cat1 protein sequence on a contig with NCBI accession code JAKEFQ010000444. Homologs were then size filtered to be between 240 and 350 residues (original Cat1 is 258 residues) to ensure homologs may possess both CARF and TIR domains. Protein sequences for these homologs were downloaded and aligned in Geneious version 2022.2.1 using the built in Clustal Omega alignment tool with default parameters. The resulting alignment file was used to generate a phylogenetic tree using the Geneious tree builder with default parameters. The resulting tree visualization was created using iTOL. For antiphage defense system associations, associated nucleotide contigs were collected from NCBI for each homolog and were scanned with DefenseFinder (51). The resulting hmmer hit files were then analyzed to determine whether complete type III CRISPR, CBASS, or Thoeris systems were present, as these systems have been shown either through this study or others to contain TIR domain-containing effectors that participate in nucleotide-based signaling.

### Bacterial growth

*Staphylococcus aureus* strain RN4220 (52) was grown in brain heart infusion (BHI) medium at 37°C, supplemented with chloramphenicol at 10 μg ml−1 for maintaining pCRISPR, and erythromycin at 10 μg ml−1 for maintaining pTarget. 5 μM CaCl_2_ was supplemented in phage experiments unless indicated otherwise.

### Growth curves

For *in vivo* Cad1 toxicity induction, biological replicates of RN4220 overnight cultures containing pTarget and pCRISPR are diluted 1:100, outgrown for about an hour and normalized for optical density. Cells are then seeded in a 96-well plate. To induce targeting, 125 ng ml^−1^ of aTc is added to the appropriate wells. Absorbance at 600 nm is then measured every 10 min by a microplate reader (BioTek Synergy H1).

For *in vivo* antiphage immunity, cells containing various pCRISPRs were launched in triplicate overnight, diluted 1:100, outgrown for about an hour and normalized for optical density. Cells were seeded into a 96-well plate. Phages were added at the specified MOIs, and optical density measurements were taken every 10 min.

### Cat1 toxicity assay

To measure the effect of Cat1 activity on *S. aureus* viability over time, colonies of *S. aureus* containing pTarget and the specified pCRISPR were launched in liquid culture overnight in triplicate. The next day, cells were diluted 1:100 and grown out for about 1 h and normalized for optical density. One aliquot was taken from each culture, and then aTc was added to induce CRISPR targeting and Cat1 activity. At each time point, cell aliquots were removed, centrifuged and resuspended in medium lacking aTc, and serial dilutions were plated on solid BHI agar plates with or without aTc. All viable cells should grow on the solid agar plates, but only targeting escapers (cells that recover owing to mutations in pTarget or pCRISPR) should form colony-forming units on plates with aTc.

### Phage plaquing and enumeration of phage plaques

To obtain plaque-forming-unit counts over time from cultures infected with phage, *S. aureus* cultures containing various pCRISPRs were launched overnight, diluted 1:100 and outgrown for about one hour. Cells in media supplemented with 5 mM CalCl_2_ were then infected with phage ΦNM1γ6-GFP at an MOI of 1, and an aliquot was taken shortly after to obtain plaque-forming units at time 0. The cultures were then incubated further, with an aliquot taken at one hour. For phage plaquing assays, indicated phage stocks were plaqued on lawns of *S. aureus* containing the indicated constructs, or cells lacking an introduced pCRISPR plasmid to measure impacts on plaquing efficiency, with 10-fold serial dilutions for every spot in a lane.

### Cat1 toxicity time-course microscopy

To monitor the effects of Cat1 toxicity dynamics in greater detail, colonies of *S. aureus* containing pTarget and the specified pCRISPRs were launched in liquid culture overnight. The next day, cells were diluted 1:200 and were loaded into a CellASIC^®^ ONIX microfluidic plate along with media containing plain BHI and media containing BHI spiked with 125 ng ml^−1^ aTc. The plate was sealed and connected to a CellASIC^®^ ONIX2 Microfluidic System for microfluidic control of cells and media. Cells were incubated in the plate for 1 h at 37°C using a Tokai HIT thermal box (Zeiss) until they were loaded onto the imaging chamber. Cells were imaged with phase contrast every 2 min for 8 h. Plain BHI was flowed over cells for the first hour and 15 min followed by BHI spiked with aTc for the remaining 6 h and 45 min. Imaging was performed in a Nikon Eclipse Ti2 motorized microscope with Perfect Focus System using a CFI60 Plan Apochromat Lambda Phase Contrast DM 100x Oil Immersion objective lens (Nikon) with a Zyla 4.2 sCMOS (Andor) camera (65 nm pixels). We used a SOLA Light Engine (Lumencor) as a laser source with laser power set to 20% with an exposure time of 10 ms. All media was flown over cells with a constant pressure of 13.8 kPa.

### Time-course fluorescence microscopy of phage-infected cultures

To visualize the dynamics of phage infection and immunity provided by Cat1, colonies of *S. aureus* containing various pCRISPRs with spacers programmed to target specified ORFs in ϕNM1γ6-GFP were launched in liquid culture overnight. The next day, cells were diluted 1:200 and were loaded into a CellASIC^®^ ONIX microfluidic plate along with media containing plain BHI supplemented with 2.5 mM CaCl2 with and without ϕNM1γ6-GFP at a titer of 1.0 × 10^7^ PFUs ml^−1^. The plate was sealed and connected to a CellASIC^®^ ONIX2 Microfluidic System for microfluidic control of cells and media. Cells were incubated in the plate for one hour at 37°C using a Tokai HIT thermal box (Zeiss) until they were loaded onto the imaging chamber. Cells were imaged with phase contrast and in a GFP fluorescence channel every 2 min for 17 h. For the first hour, BHI supplemented with CaCl2 was flowed over cells followed by 15 min of phage flowed over. Finally, BHI supplemented with CaCl2 was flowed over for the remaining 15 h and 45 min. The same phase contrast settings used in the Cat1 toxicity microscopy were used in these experiments, however, the GFP channel was measured with a C-FL GFP HC HISN Zero Shift filer (Excitation: 470/40 nm (450-490 nm), Emission: 525/50 nm (500-550 nm), Dichroic Mirror: 495 nm) (Nikon). GFP channel imaging was performed with the SOLA Light Engine set to 2% laser power with a 200-ms exposure time. All media was flown over cells with a constant pressure of 13.8 kPa.

### *In vivo* NAD(H) measurement from lysates

NAD(H) levels were measured from staphylococcal lysates using the NAD/NADH Assay Kit (colorimetric) from Abcam. To prepare lysates, 45 mL cultures were prepared with a 1/100 dilution of overnight cultures containing both pCRISPR and pTarget. These cultures were grown for an hour and were then diluted to normalize OD_600_ and induced with 125 ng/mL aTc for 30 minutes. Cells were then spun down into pellets and resuspended in 500 uL of PBS. Resuspended pellets were transferred to 1.5 mL tubes and spun down again. Supernatants were then aspirated and resuspended again in 500 uL of PBS. 50 uL of 2 mg/mL lysostaphin was then added and the solution was incubated at 37 degrees Celsius for 45 minutes. Samples were spun down again and supernatants were spun through 10 kDa cutoff filters. 25 uL of filtrates were used for final assays following manufacturers protocols.

### *In vitro* NADase reactions

NADase reactions were performed *in vitro* by incubating substrates at 37° for 2 h unless otherwise indicated. Reaction substrates were supplied at 1 mM final concentrations with Cat1 at the indicated concentrations. Additionally, ten molar equivalents of the Cat1 concentration of the indicated cOA activators were added to reaction mixes. All reactions were performed in 25 mM MES pH 6, 200 mM NaCl, 2 mM bME, and 5% glycerol. After incubations at 37°, reactions were then heated to 85° for 3 min to quench reactions and then cooled to 4° before removing from thermal blocks. Cooled reactions were diluted with nuclease-free water before being filtered with Amicon^®^ Ultra Centrifugal Filter, 3 kDa MWCO filters to remove proteins before analysis. 10 mL of final clean reaction products were then injected onto an Agilent Bonus-RP, 4.6 × 150 mm, 3.5 um Rapid Res. C18 column held at 40° at a flow rate of 1.2 mL per min. Chromatograms were collected with a mobile phase of 100/0% A/B for two minutes followed by 95/5% A/B in two minutes, then 80/20% A/B in one and a half minutes, 75/25% A/B in 30 s, and 100/0% A/B in two minutes. Buffer A is composed of 60 mM dipotassium hydrogen phosphate and 40 mM potassium dihydrogen phosphate at pH 7 and Buffer B is pure acetonitrile. Traces were collected by monitoring absorbance at 254 nm.

### In vitro ring nuclease reactions

To monitor potential ring nuclease activity by the CARF domain of Cat1, we incubated 500 mM cA4 with 2 mM full length Cat1 or cOA alone in the same reaction buffer mentioned previously. Reactions were carried out at 37° for 16 h before filtering the reaction products and collecting chromatograms. Chromatograms were collected with a mobile phase of 100/0% A/B for two minutes followed by 95/5% A/B in two minutes, then 80/20% A/B in one and a half minutes, 75/25% A/B in 30 s, and 100/0% A/B in two minutes. Buffer A is composed of 60 mM dipotassium hydrogen phosphate and 40 mM potassium dihydrogen phosphate at pH 7 and Buffer B is pure acetonitrile.

### Heterologous expression and purification of Cat1-His_6_.

The construct containing Cat1-His6 and its mutants were expressed in *Staphylococcus aureus* RN4220 cells using Difco^™^ Brain Heart Infusion media (BD). Cells were grown at 37 degrees Celsius to an OD600 of 0.7 at which expression was induced with the addition of 1 mM IPTG. Cells were then incubated with shaking at 18 degrees Celsius overnight. The cells were harvested by centrifugation at 4k rpm and resuspended in the lysis buffer (25 mM MES pH 6, 500 mM NaCl, 2 mM β-Mercaptoethanol and 5 % glycerol) supplemented with cOmplete mini, EDTA-free protease inhibitor tablets (Sigma). 5 mg of Lysostaphin (Sigma) was used to degrade the cell walls of the cells harvested from a 6 L culture by incubating Lysostaphin and the resuspended cells for 1 hour at 4°C. Next, the cells were lysed using sonication at 50 % amplitude for total 15 minutes with 1 s on and 2 s off pulse. The unlysed cell debris were separated from the cell lysate by centrifugation at 22 k rpm for 1 hour. The clear cell lysate was passed through the pre-equilibrated (with lysis buffer) 5 ml HisTrap column (Cytiva). The protein was eluted using the lysis buffer supplemented with 300 mM imidazole. The elution fractions were loaded to NuPAGE^™^ 4-12 % Bis-Tris gel (invitrogen) and pure protein fractions were pulled together and concentrated to 500 μl. The concentrated protein was loaded to Superose 6 10/300-increase column (Cytiva) pre-equilibrated in buffer A (25 mM MES pH 6, 200 mM NaCl, 2 mM β-Mercaptoethanol and 5 % glycerol). Pure fractions were collected for subsequent studies. The DN158, DN103-A104, D33A and Y122A mutant constructs were purified using the method described above. The apo wild type Cat1, K225A-S235A, R227A- W215A, K225A-R227A-W215A-S235A mutant constructs were purified by the same method and size column chromatography was performed using Superdex 200 10/300-increase column (Cytiva).

### Isothermal Titration Calorimetry (ITC) binding studies

ITC binding experiments were performed with 40 μM of Cat1(K225A-S235A)-His6 protein titrated with 400 μM of cA4. The protein and the ligand were present in 25 mM MES pH 6, 200 mM NaCl, 2 mM β-mercaptoethanol and 5 % glycerol. MicroCal PEAQ-ITC (Malvern) instrument was used to perform the ITC studies at 25° C temperature. The protein was titrated with a total of 19 injections of cA4 out of which the volume of the first injection was 0.4 μl with a 0.8 s duration and rest of the 18 injections were 2 μl with a 4 s duration. The spacing between each of the injections was 150 s and the stirring speed was 750 rpm. The data was analyzed with MicroCal PEAQ-ITC Analysis Software (Malvern). The NDH_X and NDH_Y values estimated by MicroCal PEAQ-ITC Analysis Software (Malvern) is represented using GraphPadPrism version 10.3.0.

### SEC-MALS analysis of Cat1-His6 protein in presence of NAD^+^

To determine the oligomeric state of Cat1-His6 in the presence of NAD^+^ (1:10 molar ratio) the peak fractions were pulled from the previously described size exclusion chromatography run. The SEC-MALS experiment was performed using an AKTA-Pure UV detector connected to SEC-MALS instrument with multi-angle light scattering detector and refractive index detector (Wyatt). The pure protein was loaded onto a Superose 6 10/300-increase column (Cytiva) preequilibrated with 25 mM MES pH 6, 200 mM NaCl, 2 mM β-mercaptoethanol and 5 % glycerol. ASTRA 6 software was used for data analysis. The UV signal of the AKTA-Pure instrument was converted to analogue signal with a conversion factor of 1,000 mAU = 1 V. For the analysis the refractive index, the increment (dn/dc) value was 0.185 ml g^−1^ for Cat1-His6.

### CryoEM sample preparation and imaging

The purified apo-Cat1 protein was concentrated to 40 μM using 30 kDa cutoff Amicon Ultra Centrifugal Filter (Millipore Sigma) and used for grid preparation. In the case of the cA4- Cat1 grid preparation 83 μM of cA4 was added to 40 μM of apo-Cat1 protein and the grids were frozen instantly with 10 s. In the case of cA4-Cat1-BAD and cA4-Cat1-NAD grid preparation, 40 μM of apo-Cat1 protein was first incubated with 1 mM BAD or 1 mM of NAD respectively for 30 minutes. Further, 83 μM of cA4 was added to the Cat1-BAD or Cat1-NAD preincubated sample and grids were frozen within 10 s of cA4 addition as described previously. We used QuantiFoil Au R (1.2/1.3) 300 mesh grids for all the samples. The grids were glow-discharged for 2 minutes at 15 mA prior freezing. The grids were frozen at 100 % humidity, 10 s wait time, 3 s blot time and +6 blot force at 4 °C temperature using a Vitrobot Mark IV (FEI). The cA4- Cat1 dataset was collected on a Titan Krios equipped with a Falcon IV detector and an energy filter with a slit width of 20 eV at NCCAT (National Center for CryoEM Access and Training). The dataset was collected at a pixel size of 0.73 Å and the images were recorded at a defocus range of −0.8 μm to −2.0 μm. The images were collected in the EER mode with a total electron dose of 45 electrons per Å^2^ with an exposure time of 6 s. The EER upsampling factor was 1 and EER number of fractions were 60. The cA4-Cat1-BAD dataset was collected at Simons Electron Microscopy Center at MSKCC using a Titan Krios G4 connected with a Falcon 4i camera and an energy filter of 10 eV slit width. The pixel size was 0.73 Å and the dataset was recorded at a defocus range of −0.8 μm to −2.3 μm. The images were collected in the EER mode with a total electron dose of 59.33 electrons per Å^2^ with an exposure time of 2.9 s. The EER upsampling factor was 1 and EER number of fractions were 45. cA4-Cat1-BAD dataset was collected on a Titan Krios equipped with a Gatan K3 camera and an energy filter with 20 eV slit width. The images were acquired with 0.856 Å pixel size and at a defocus range of −1 μm to −2.5 μm. The total electron dose for the data collection was 57.32 electrons per Å^2^ and the exposure time was 2.5 s fractioned over 50 movie frames.

### CryoEM data processing and refinement

We used cryoSPARC v4.4.1 for the processing of the cA4-Cat1, cA4-Cat1-BAD and cA4- Cat1-NAD datasets (53). Data collection and refinement parameters are provided in Table S1.

#### Processing of cA4-Cat1 dataset.

We imported 10,749 dose weighted micrographs to cryoSPARC and performed patch CTF estimation. Next, we manually picked 2,323 particles from 138 micrographs and performed 2D classification. From the 2D classification job nine 2D classes correspond to 2,191 particles resembled filaments were further chosen as templates for template picker job. 12,213 particles correspond to good 2D classes were chosen by iterative 2D classification and used for Topaz training job. 3,068,364 initial particles were picked by Topaz extract job and particles were extracted with 512 box size. Next, we used 49,051 particles to build an ab-initio model. 2,746,966 particles were selected by iterative 2D classification and used for Hetero-refinement job and selected 921,289 particles. Next, we removed 536,289 duplicate particles and used 385,000 particles for 3D classification job. The 3D volumes were classified into four classes with 127,170 particles in class 1; 116,684 particles in class 2; 84,493 in class 3 and 56,653 particles in class 4. Out of which class 3 corresponded to triagonal filament bundle and the map was refined to 3 Å resolution by non-uniform refinement job following the standard FSC cutoff value of 0.143. The particles correspond to class 4 were further classified into 3 classes. Out of which we selected class 2 with 12,721 particles correspond to pentameric filament bundle and refined to 3.4 Å resolution using non-uniform refinement job following the standard FSC cutoff value of 0.143 (Fig. S8). The AlphaFold3 (54) model of the CARF and the TIR domains were fitted to the maps using Chimera (55) and the models was built using Coot (56). The density of the cA4 signaling molecules were well resolved and the cA4 molecule could be fitted with confidence (Figs. S9A–B). Phenix real-space refinement program was used to remove the outliers and refine the models with a model vs. data correlation value (CC mask) of 0.89 and 0.86 for the triagonal filament bundle and pentameric filament bundle respectively.

#### Processing of cA4-Cat1-BAD dataset.

8,318 movies were collected, and patch motion correction was performed using cryoSPARC. Patch CTF estimation job was performed with the motion corrected micrographs. 1439 particles were selected by 2D classification out of 2010 manually picked particles and 13,238 particles were selected similarly from 306,287 particles picked by blob picker from 1,220. The extraction box size was 512 pixel (0.73 Å/pixel). Next, the selected particles described above were used to train the picking model in Topaz train job. 3,552,142 particles were picked up and extracted by topaz extract and particle extract job respectively with 512 box size. We used 20,660 particles for building the ab-initio model. Iterative 2D classification job was used to select 958,227 particles and used as input to heterorefinement job. 570,989 particles were selected by hetero-refinement and used as input in the 3D classification job. The particles were classified in seven 3D classes. Out of which 120,866 particles were selected corresponding to class 1 and duplicate particles were removed. The accepted 87,475 particles were further classified into 3 classes using 3D classification job. Out of which class 1 and class 2 with 45,402 and 42,056 particles were merged and refined to 3.3 Å resolution by non-uniform refinement job following the standard FSC cutoff value of 0.143 (Fig. S10). The AlphaFold3 (54) model of the CARF and the TIR domains were fitted to the map using Chimera (55) and the models was built using Coot (56). The density of the cA4 signaling molecules were well resolved and the cA4 molecule could be fitted with confidence (Fig. S9C). The density of the BAD was poorly resolved and model fitting is displayed in Figure. S9E (left panel). Phenix real-space refinement program was used to remove the outliers and refine the model with a model vs. data correlation value (CC mask) of 0.85.

#### Processing of cA4-Cat1-NAD^+^ dataset.

7,486 dose weighted micrographs were imported to cryoSPARC and patch CTF estimation was performed. We used blob picker to pick up 978,943 particles from 2538 micrographs and the particles were extracted with 512 box size (0.856/pixel). 28,686 particles were selected by 2D classification job for ab-initio model building. The same set of particles described above was used to train the picking model by topaz training job. 1,941,502 particles were picked and extracted by topaz extract and particle extraction job respectively. 458,188 particles were selected by 2D classification job and used as input to hetero-refinement job. Further, 230,728 particles were selected by hetero- refinement job and classified into five classes by 3D classification job. Out of which 57,560 particles correspond to class 2 were selected and duplicates particles were removed. The accepted 48,074 particles were used for train particle picking model by topaz train job. 1,137,652 particles were picked and extracted by topaz extract and particle extraction job respectively. 2D classification was used to select 596,288 particles and used as input to hetero-refinement job and classified into 10 classes. Out of which class 1 was selected having 152,949 particles. These particles were further cleaned up by hetero-refinement job and finally 152,750 particles were selected belonging to class 1 and refined to 4 Å resolution by non-uniform refinement job following the standard FSC cutoff value of 0.143 (Fig. S11). The AlphaFold3 (54) model of the CARF and the TIR domains were fitted to the map using Chimera^47^ and the models was built using Coot (56). The density of the cA4 signaling molecules were well resolved and the cA4 molecule could be fitted with confidence (Fig. S9D). The density of the ADPR was poorly resolved and model fitting is displayed in Fig. S9E (right panel). Phenix real-space refinement program was used to remove the outliers and refine the model with a model vs. data correlation value (CC mask) of 0.86.

## Supplementary Material

Table S1

Supplementary materials

Data S1

Data S2

## Data Availability

All data are included in the manuscript or supplement. Cryo-EM maps have been deposited in the Electron Microscopy Data Bank (EMDB) under the accession codes EMD-48698 (Cat1-cA4 trigonal filament assembly), EMD-48629 (Cat1- cA4 pentagonal filament assembly), EMD-48630 (Cat1-cA4-NAD pentagonal filament assembly), EMD-48639 (Cat1-cA4-BAD pentagonal filament assembly). The corresponding atomic coordinates of the cryo-EM structures have been deposited in the Protein Data Bank (PDB) under the accession codes 9MW9 (Cat1-cA4 trigonal filament assembly), 9MUD (Cat1-cA4 pentagonal filament assembly), 9MUE (Cat1-cA4-NAD), 9MUO (Cat1-cA4- BAD). All Cat1 homologs (along with their NCBI protein accession code, associated amino acid sequences, sequence lengths, and organism of origin) collected for bioinformatic analysis are included in Data S1. Additionally, associated Cas10 sequences and the presence or absence of a catalytic HD domain for each of these proteins is reported in the same file. Catalytic HD domains were determined using HMMscan with pfam model PF01966.28 using default parameters. Any additional information required to reanalyze the data reported in this paper is available from the lead contact upon request.
