# Supplementary material for: Cat1 forms filament networks to degrade NAD+ during the type III CRISPR- Cas anti-viral response
Source: Science. Author manuscript; Available in PMC 2025 Jun 13. (PMC12162218; doi:10.1126/science.adv9045)
Supplement: Table S1 [file NIHMS2075442-supplement-Table_S1.docx]

**Cryo-EM data collection, refinement, and validation statistics**

|  | cA_4_-Cat1 (Trigonal)  (PDB 9MW9)  (EMDB 48698) | | cA_4_-Cat1  (Pentagonal)  (PDB 9MUD)  (EMDB 48629) | cA_4_-Cat1-BAD  (Pentagonal)  (PDB 9MUO)  (EMDB 48639) | cA_4_-Cat1-NAD  (Pentagonal)  (PDB 9MUE)  (EMDB 48630) |
| --- | --- | --- | --- | --- | --- |
| **Data collection and Processing (for each dataset)** |  | |  |  |  |
| Microscope  Voltage (keV)  Camera  Magnification  Pixel size at detector (Å/pixel)  Total electron exposure (e^–^/Å^2^)  Exposure rate (e-/pixel/sec)  Number of frames collected during exposure  Defocus range (μm)  Phase plate (if used)  - phase shift range (in degrees)  - number of images per phase plate position  Automation software (EPU, SerialEM or manual)  Tilt angle (if grid was tilted)  Energy filter slit width (eV)  Micrographs collected (no.)  Micrographs used (no.)  Total extracted particles (no.)  **For each reconstruction:**  Refined particles (no.)  Final particles (no.)  Point-group or helical symmetry parameters  Estimated error of translations/rotations (if available)  Resolution (global, Å)  FSC 0.5 (unmasked/masked)  FSC 0.143 (unmasked/masked)  Resolution range (local, Å)  Resolution range due to anisotropy (Å)  Map sharpening *B* factor (Å^2^) / (B factor Range)  Map sharpening methods | | Titan Krios  300  Falcon IV  165000  0.73  45  4  60  -0.8 to -2  -  -  -  Leginon (NCCAT)  -  20  10,749  10,749  3,068,364  84,493  84,493  -  -  3  -  3.8/3  3-7  -  -  - | Titan Krios  300  Falcon IV  165000  0.73  45  4  60  -0.8 to -2  -  -  -  Leginon (NCCAT)  -  20  10,749  10,749  3,068,364  12,721  12,721  -  -  3.4  -  4.7/3.4  3.4-9  -  -  - | Krios G4  300  Falcon 4i  165000  0.725  59.33  11.5  45  -0.8 to -2.3  -  -  -  EPU  -  10  8,318  8,318  3,552,142  87,458  87,458  -  -  3.3  -  4/3.3  3.3-7  -  -  - | Titan Krios  300  Gatan K3  81000  0.856  57.32  20  50  -1 to -2.5  -  -  -  Leginon (NCCAT)  -  20  7,486  7,486  1,941,502  152,750  152,750  -  -  4  -  4.3/4  3.6-8  -  -  - |
|  |  | |  |  |  |
| **Model composition (for each model)**  Protein  Ligands  RNA/DNA  **Model Refinement (for each model)** | 5478  0  44 | | 7470  0  60 | 1012  1  8 | 1012  2  8 |
| Refinement package  - real or reciprocal space  - resolution cutoff  Model-Map scores  -CC | Phenix  Real space  3  0.89 (mask) | | Phenix  Real space  3.4  0.86 (mask) | Phenix  Real space  3.3  0.85 (mask) | Phenix  Real space  4  0.86 (mask) |
| - Average FSC (d FSC at 0.143) | 3.1 | | 3.7 | 3.4 | 4 |
| *B* factors (Å^2^) |  | |  |  |  |
| Protein residues | 167.64 | | 173.70 | 130.28 | 120.32 |
| Ligands  RNA/DNA | -  123.42 | | -  135.63 | 214.76  58.10 | 155.45  84.98 |
| R.m.s. deviations from ideal values |  | |  |  |  |
| Bond lengths (Å) | 0.004 | | 0.003 | 0.004 | 0.004 |
| Bond angles (°)  **Validation (for each model)**  MolProbity score  CaBLAM outliers  Clashscore  Poor rotamers (%)  C-beta deviations  EMRinger score (if better than 4 Å resolution)  Ramachandran plot  Favored (%)  Outliers (%) | 0.759  2.1  2.07  12.84  2.01  0  -  96.25  0.00 | | 0.720  1.98  2.34  15.77  0.47  0  -  95.96  0.00 | 0.774  2.02  3.04  13.49  0.67  0  -  94.40  0.20 | 0.716  2.12  4.25  16.02  0.11  0  -  93.80  0.10 |
