# Supplementary material for: Cat1 forms filament networks to degrade NAD+ during the type III CRISPR- Cas anti-viral response
Source: Science. Author manuscript; Available in PMC 2025 Jun 13. (PMC12162218; doi:10.1126/science.adv9045)
Supplement: Supplementary materials [file NIHMS2075442-supplement-Supplementary_materials.pdf]

Science
AAAS

Supplementary Materials for

Cat1 forms filament networks to degrade NAD^+ during the type III CRISPR-Cas anti-viral response

Christian F. Baca, Puja Majumder, James H. Hickling, Dinshaw J. Patel, Luciano A. Marraffini

Corresponding authors: majumdp@mskcc.org, pateld@mskcc.org, marraffini@rockefeller.edu

The PDF file includes:

Materials and Methods
Figs. S1 to S11
Table S1
References

Other Supplementary Materials for this manuscript include the following:

Data S1
Data S2

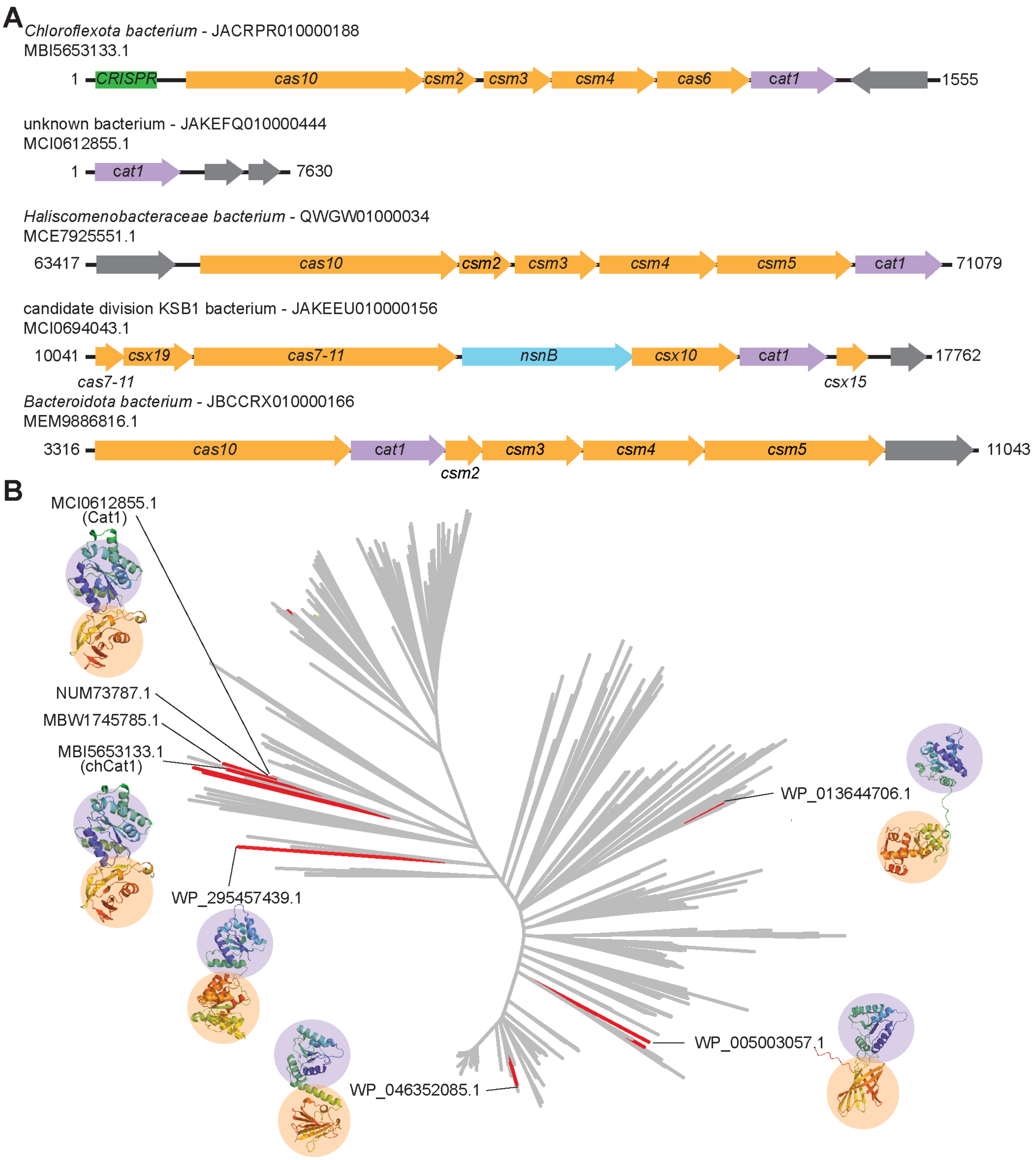

Fig. S1.

Phylogenetic analysis of Cat1 homologs. (A) Representative type III CRISPR-*cas* loci harboring *cat1* homologs (purple). The organism, contig nucleotide accession code and Cat1 protein accession number are indicated. Nucleotide positions are provided on the left and right of each locus. Orange arrows, type III cas genes; green box, CRISPR array. **(B)** Unrooted phylogenetic tree built with Cat1 homologs collected with a PSI-BLAST search. Nucleotide contigs in which Cat1 homologs were found were collected and scanned for defense systems

using DefenseFinder[43]. Branches colored in red indicate the presence of a type III CRISPR-Cas system within the Cat1-containing contig. Representative Cat1 protein sequences from different clades were used to obtain Alphafold3[44] structure predictions, and their protein accession codes are provided.

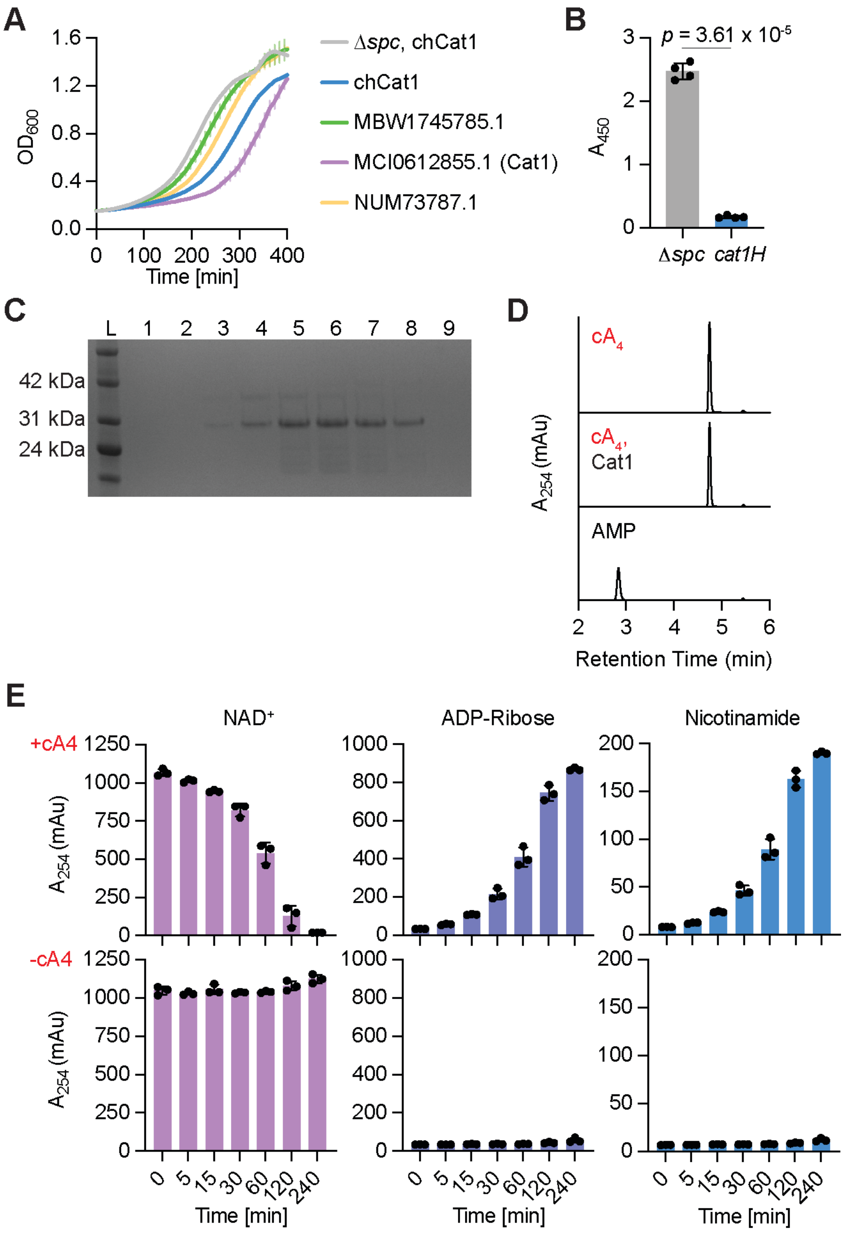

Fig. S2.

Cat1 characterization. (A) Growth of staphylococci carrying pTarget and pCRISPR harboring different *cat1* homologs (some noted by their protein accession codes), measured as OD_{600} after the addition of aTc. Mean of three biological triplicates, ±s.e.m., is reported. **(B)** NAD(H) measurement in lysates of staphylococci harboring the indicated pCRISPR constructs and pTarget, 30 minutes after aTc addition. Absorbance at 450 nm was measured in a colorimetric assay. Four biological triplicates with ±s.e.m are reported. **(C)** Coomassie-blue SDS-PAGE of elution fractions (numbered) collected during Ni-NTA affinity chromatography of Cat1-His_6. **(D)** Testing cleavage of cA_4 by Cat1. cA_4 (500 mM) was incubated for 16 hours at 37 °C in the absence or presence of Cat1 (2 mM). Reactions were then treated for HPLC separation of the substrate and the reaction products with absorbance at 254 nm as the readout. Adenosine

monophosphate (AMP) was included as a standard. **(E)** Time-course reaction kinetics of Cat1 in the absence and presence of cA_4. Reactions were performed with 2 mM Cat1 and 20 mM cA_4; incubated at 37 °C for the indicated amount of time (0-240 minutes), and then treated for HPLC separation of the substrate and the reaction products with absorbance at 254 nm as the readout.

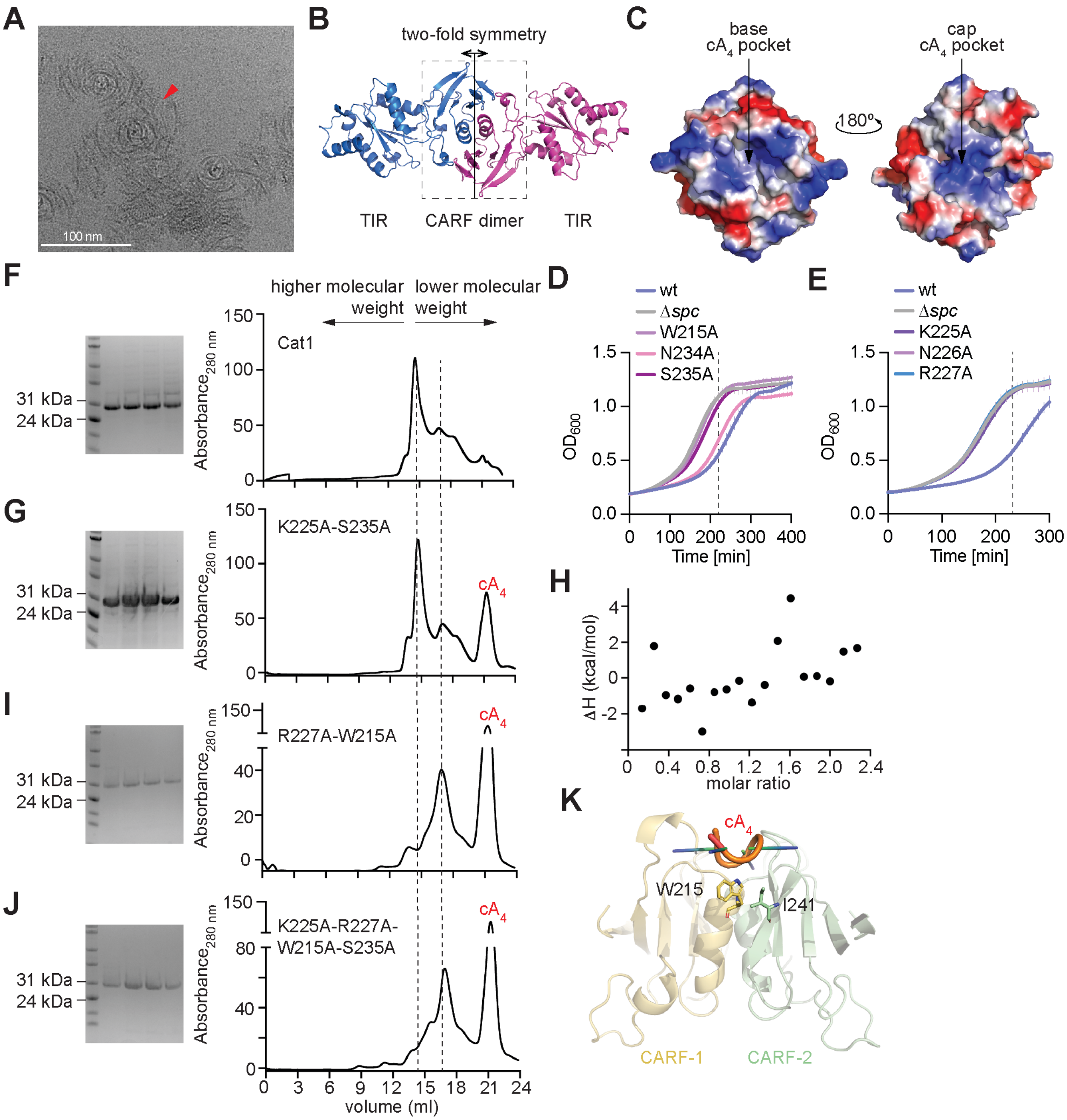

Fig. S3.

Structural details of Cat1's cA4 binding pocket. (A) Representative micrograph displaying cA_4-Cat1-His_6 filaments indicated by a red arrowhead; the scale bar is 100 nm. **(B)** Cat1 filament repeat unit displaying two-fold symmetry between Cat1 monomers, colored in blue and magenta. **(C)** Surface charge representation of the dimeric CARF domain of a Cat1 filament displaying the base (left) and the cap of the cA_4 binding pocket (right). **(D)** Growth of staphylococci carrying pTarget and pCRISPR expressing Cat1 mutants with different alanine substitutions in residues that form the base of the cA_4 binding pocket, measured as OD_{600} after the addition of aTc. Dashed line; timepoint at 220 minutes used to obtain OD_{600} values for bar graphs. Mean of three biological triplicates, ±s.e.m., is reported. **(E)** Same as **(D)** but testing the expression of Cat1

mutants with different alanine substitutions in residues that form the cap of the cA_4 binding pocket. **(F)** Coomassie-stained SDS-PAGE of purified apo wild-type Cat1 protein (left) followed by size exclusion chromatography, which shows the formation of a dimer. **(G)** Same as **(F)** but for purified Cat1 harboring alanine mutations in both K225 (cap of the cA_4 pocket) and S235 (base of the cA_4 pocket), in the presence of cA_4. The elution peak (same as apo wild-type Cat1) indicates that the mutant protein does not form filaments and stays as a dimer. **(H)** ITC binding study of Cat1 K225A-S235A mutant to cA_4 representing the NDH_X and NDH_Y values estimated by MicroCal PEAQ-ITC analysis software (Malvern). **(I)** Same as **(F)** but for purified Cat1 harboring alanine mutations in both R227 (cap of the cA_4 pocket) and W215 (base of the cA_4 pocket), in the presence of cA_4. The shift of the elution peak indicates that the mutant protein not only does not form filaments but also is unable to dimerize, most likely eluting as a monomer. **(J)** Same as **(I)** but for purified Cat1 harboring all four mutations, K225A-R227A-W215A-S235A, in the presence of cA_4. The shift of the elution peak indicates that the mutant protein not only does not form filaments but also is unable to dimerize, most likely eluting as a monomer. **(K)** Potential hydrophobic interaction between W215 of one CARF domain with I241 of the adjacent CARF domain. The interaction suggests that, most likely, the W215A mutation disrupts Cat1 dimerization.

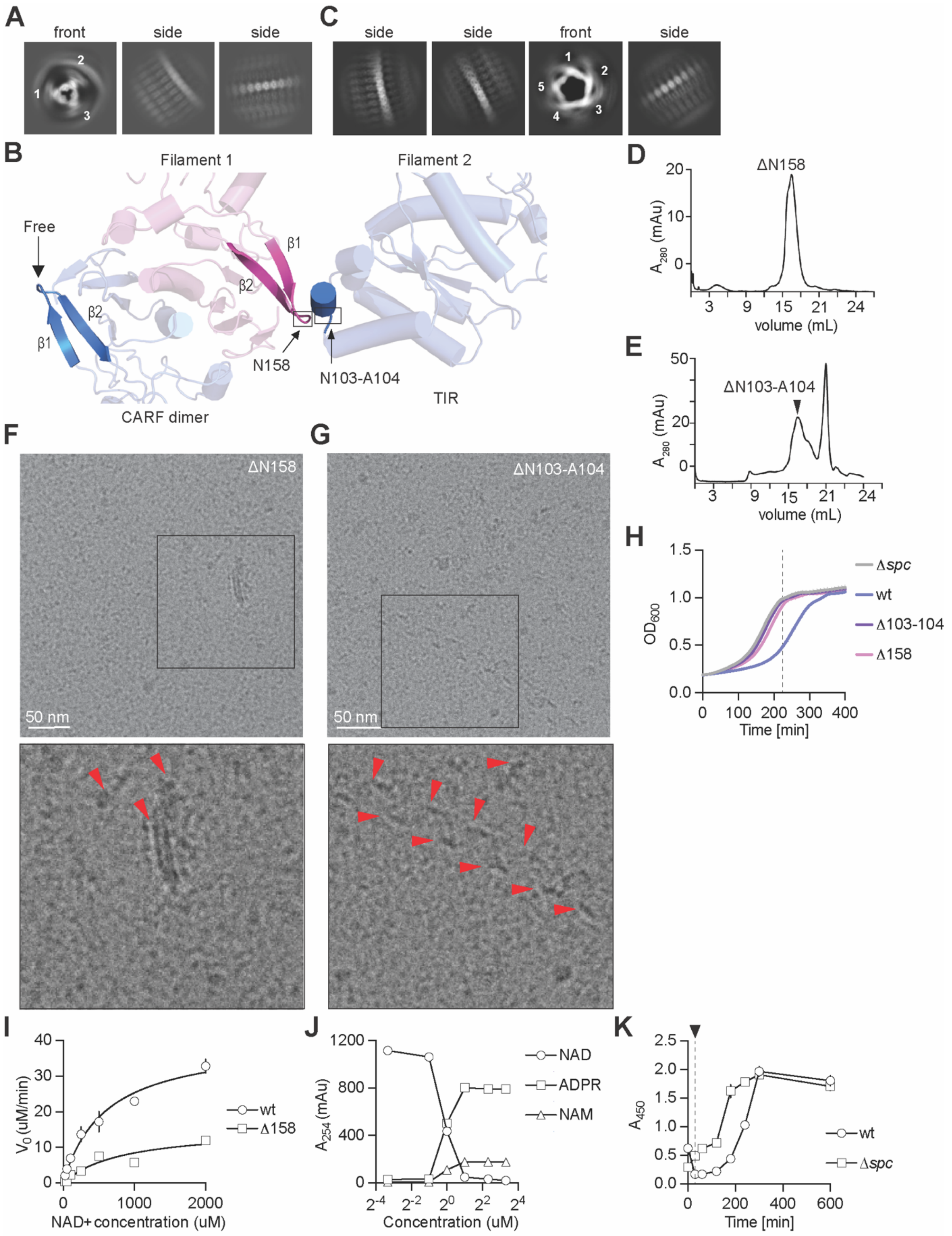

Fig. S4.

Characterization of Cat1's inter-filament interactions. (A) Representative 2D class averages with 40k particles displaying the front and the side views of the trigonal filament assembly of the

cA_4-Cat1 complex. Each of the three filaments are indicated by numbers (1-3) in the image of the front view. The side views are showing the arrangement of the repeat units. **(B)** Magnified view of the inter-repeat unit interaction between the β1β2-loop of the CARF domain of one Cat1 dimeric unit and the N103-A104 loop of the TIR domain of the adjacent Cat1 dimer. **(C)** The front and side views of 2D class averages with 40 k particles showing the pentameric filament assembly of cA_4-Cat1 complex. Each of the five trigonal filaments are indicated by numbers (1-5) in the image of the front view. **(D)** Size exclusion chromatogram of purified Cat1-His_6(ΔN158). **(E)** Size exclusion chromatogram of purified Cat1-His_6(ΔN103-A104). **(F)** Representative micrograph of purified Cat1-His_6(ΔN158) collected at 165000x magnification. The black border points to the zoomed in region shown in the bottom inset. Small filament fragments are pointed by red arrowheads. Scale bar, 50 nm. **(G)** Same as **(F)** for purified Cat1-His_6(ΔN103-A104). **(H)** Growth of staphylococci carrying pTarget and pCRISPR expressing different deletions of Cat1 residues involved in inter-filament interactions, measured as OD_{600} after the addition of aTc. Dashed line; timepoint at 220 minutes used to obtain OD_{600} values for bar graphs. Mean of three biological triplicates, ±s.e.m., is reported. **(I)** Measurement of reaction kinetics for Cat1 wild-type and Δ158. Reactions were performed with 500 nM Cat1, 1 mM NAD+, and 5 mM cA_4 for ten minutes at 37 °C, then treated for HPLC separation of the substrate and the reaction products with absorbance at 254 nm as the readout. Calculated V_{max} values for wild-type and Δ158 are 40.18 and 15.38 mM/min, respectively. Calculated K_m values for wild-type and Δ158 are 585.8 and 821.7 mM, respectively. Reactions were performed in triplicates, ±s.e.m., is reported. **(J)** Measurement of NAD^+ cleavage using different Cat1 concentrations. NAD^+, 1 mM; cA_4, 10 mM. Reactions were incubated at 37 °C for 30 minutes, then treated for HPLC separation of the substrate and the reaction products with absorbance at 254 nm as the readout. Reactions were performed in triplicates, ±s.e.m., is reported. **(K)** Measurement of cellular NAD(H) overtime after a burst of cA_4. Cells with the indicated pCRISPR and pTarget were grown for one hour and normalized to OD_{600} of 0.24. NAD(H) levels were measured for time zero and then 125 ng/mL aTc was added. 30 minutes after the addition of aTc, cellular NAD(H) levels were measured again and the cultures were then washed with equivalent volumes of media lacking aTc twice (arrowhead and dotted line). Cellular NAD(H) levels were then measured 60, 120, 180, 240, 300 and 600 minutes after the initial innoculation. All measurements came from the same amount of cells, normalized by OD_{600}. Mean of three biological triplicates, ±s.e.m., is reported.

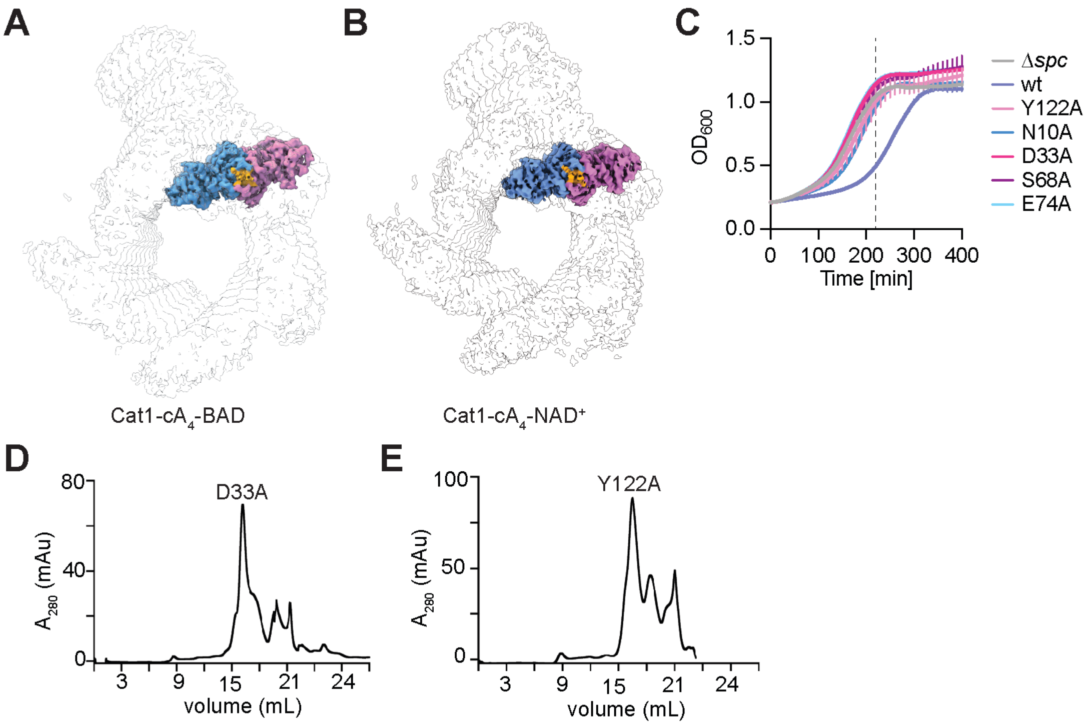

Fig. S5.

Structural details of the NADase catalytic pocket of Cat1 filaments. (A) Cryo-EM map of the pentameric helical filament bundle of the cA_4-BAD-Cat1-His_6 complex. A pair of Cat1 dimers used for building the active site model is shown, with blue and magenta Cat1 monomers. **(B)** Same as **(A)** for the cA_4-NAD^+-Cat1-His_6 complex. **(C)** Growth of staphylococci carrying pTarget and pCRISPR expressing Cat1 mutants with different alanine substitutions in residues that form the NADase active site, measured as OD_{600} after the addition of aTc. Dashed line; timepoint at 220 minutes used to obtain OD_{600} values for bar graphs. Mean of three biological triplicates, ±s.e.m., is reported. **(D)** Size exclusion chromatogram of purified Cat1-His_6(D33A). **(E)** Size exclusion chromatogram of purified Cat1-His_6(Y122A).

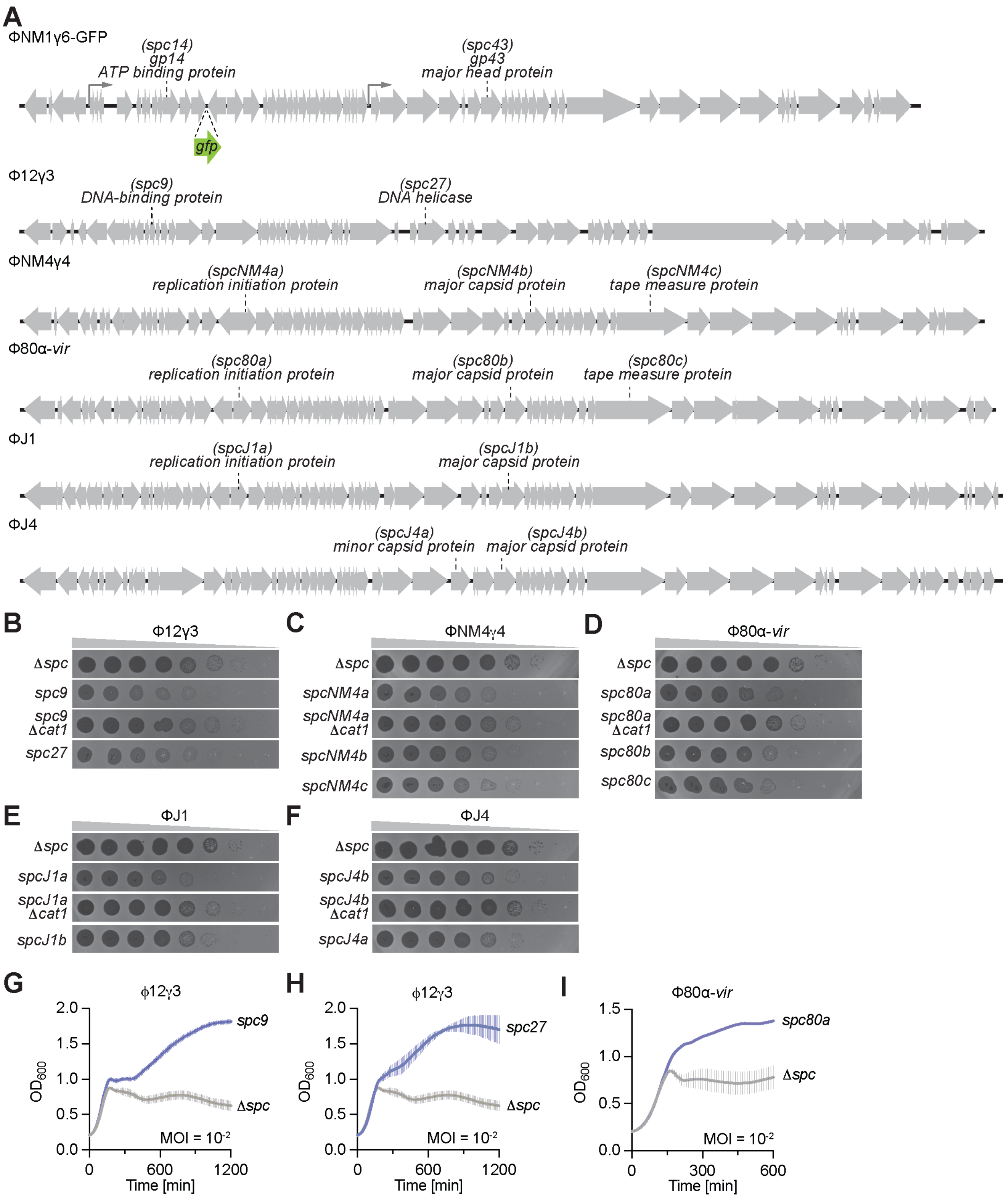

Fig. S6.

Cat1 immunity against different staphylococcal phages. (A) Schematic of the genome of the staphylococcal phages used in this study, showing the location of the transcripts targeted by the different spacers cloned into the CRISPR array of type III-A CRISPR-Cas system expressed by pCRISPR. For the ϕNM1γ6-GFP phage, the insertion site of the *gfp* gene, as well as the early

and late promoters (gray arrows), are shown. **(B-F)** Plaquing of the different phages shown in **(A)** on *S. aureus* lawns harboring pCRISPR(*cas10*^{HD}) programmed with spacers to specifically target each phage [also shown in **(A)**], in the presence or absence of Cat1 (Δ*cat1*), or a non-targeting control (Δ*spc*). Images are representative of one of three biological triplicates. **(G-I)** Growth of staphylococci carrying pCRISPR constructs programmed with spacers targeting different phages or a non-targeting spacer (Δ*spc*), measured as OD_{600} of the cultures after infection at the indicated MOI. Mean of three biological triplicates, ±s.e.m, is reported.

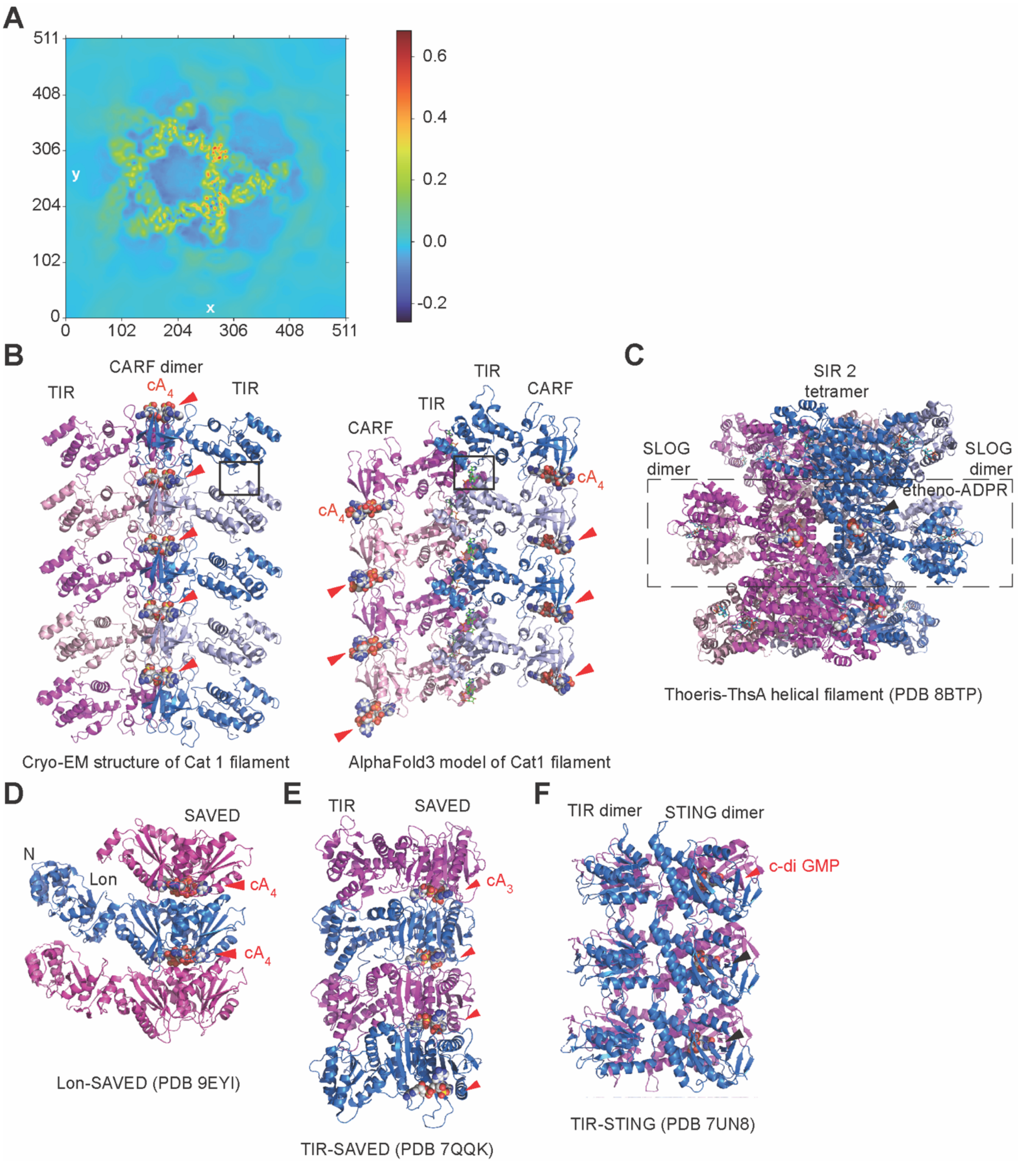

Fig. S7.

Comparison of Cat1 assembly to other anti-phage immune effectors that form filaments. **(A)** Cross section view of the xy-plane of the cryo-EM map displays the spread of the filament network of Cat1. **(B)** Cryo-EM structure of Cat1 filament (left panel) and AlphaFold3 predicted model of Cat1 filament (right panel). **(C)** Structure of ThsA helical filament (PDB 8BTP) from the Thoeris antiphage system. The ThsA filament repeat unit formed by a tetrameric SIR2 domain and two dimeric SLOG domains is marked by dashed box. **(D)** Structure of the CRISPR-

associated Lon-SAVED effector (PDB 9EYI). **(E)** Structure of the CRISPR-associated TIR-SAVED effector (PDB 7QQK). **(F)** Single filament structure of the CBASS-associated TIR-STING (PDB 7UNB) effector.

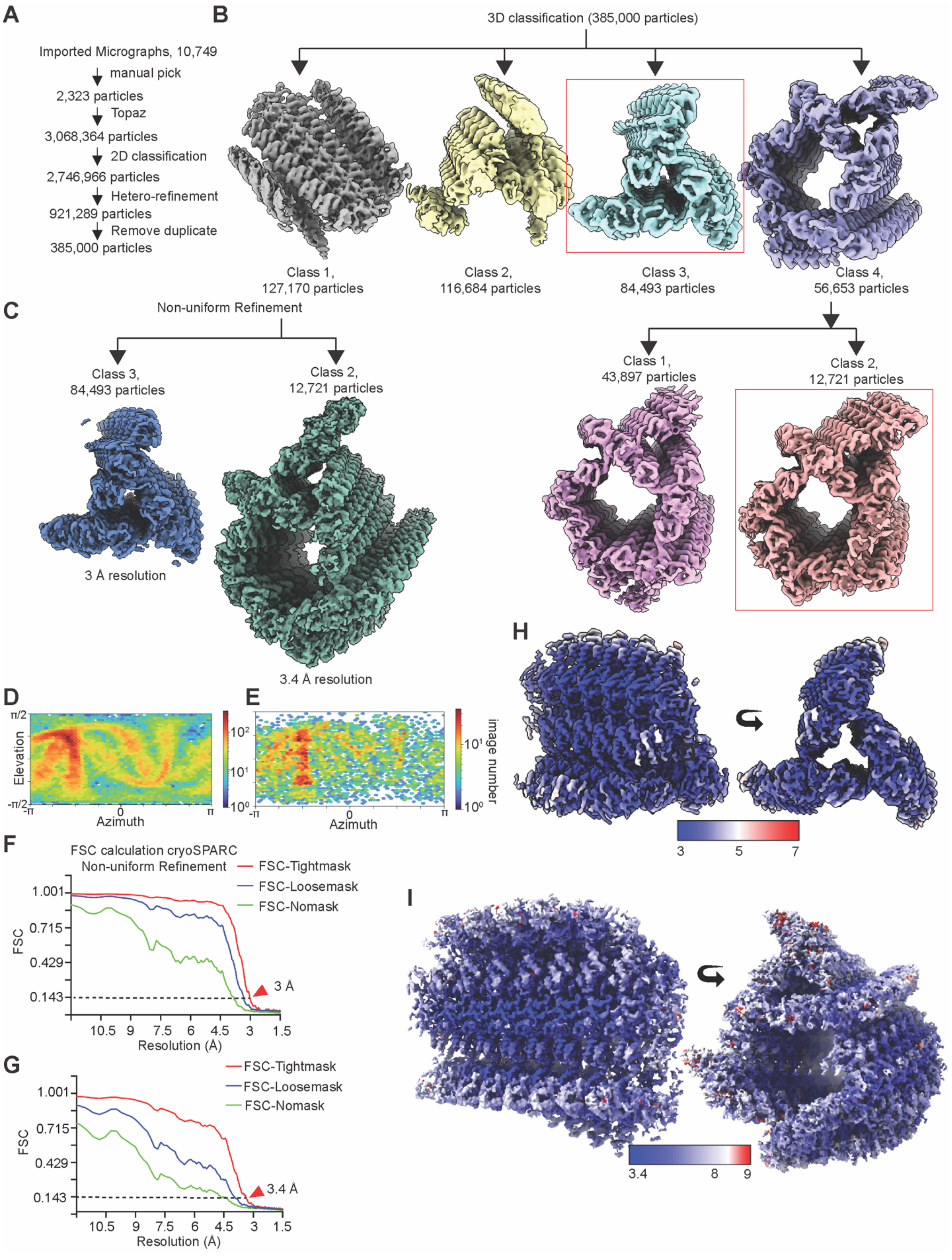

A
Imported Micrographs, 10,749
manual pick
2,323 particles
Topaz
3,068,364 particles
2D classification
2,746,966 particles
Hetero-refinement
921,289 particles
Remove duplicate
385,000 particles
B
3D classification (385,000 particles)
Class 1, 127,170 particles
Class 2, 116,684 particles
Class 3, 84,493 particles
Class 4, 56,653 particles
C
Non-uniform Refinement
Class 3, 84,493 particles
Class 2, 12,721 particles
3 Å resolution
3.4 Å resolution
Class 1, 43,897 particles
Class 2, 12,721 particles
D
Elevation
Azimuth
E
Azimuth
image number
F
FSC calculation cryoSPARC
Non-uniform Refinement
FSC-Tightmask
FSC-Loosemask
FSC-Nomask
FSC
Resolution (Å)
3 Å
G
FSC-Tightmask
FSC-Loosemask
FSC-Nomask
FSC
Resolution (Å)
3.4 Å
H
I

Fig. S8.

Cryo-EM data processing workflow of the cA_4-Cat1 structure determination. (A) cA_4-Cat1 data processing workflow is presented with the number of selected particles mentioned after each step. **(B)** The cryo-EM maps of four 3D classes obtained by 3D classification job are displayed. The particles belong to class four was classified further into two classes. The particle number correspond to each class is mentioned. **(C)** The 3D maps marked by red border in **(B)** were refined by non-uniform refinement job to 3 Å and 3.4 Å resolution for the triagonal filament assembly and pentagonal filament assembly respectively. **(D)** Angular distribution of the particles used for the generation of cA_4-Cat1 trigonal filament map. **(E)** Angular distribution of the particles used for the generation of cA_4-Cat1 pentagonal filament map. **(F)** The Fourier shell correlation (FSC) curves estimated by non-uniform refinement job with tight mask, loose mask and no mask are plotted and the resolution at 0.143 FSC value is indicated by red arrowhead for the triagonal filament assembly map. **(G)** Similar representation as **(F)** for the pentagonal filament assembly map. **(H)** The local resolution estimated by cryoSPARC local resolution estimation job for the triagonal filament assembly map. The scale bar is presented in Å unit. **(I)** Similar representation as **(H)** for the pentagonal filament assembly map.

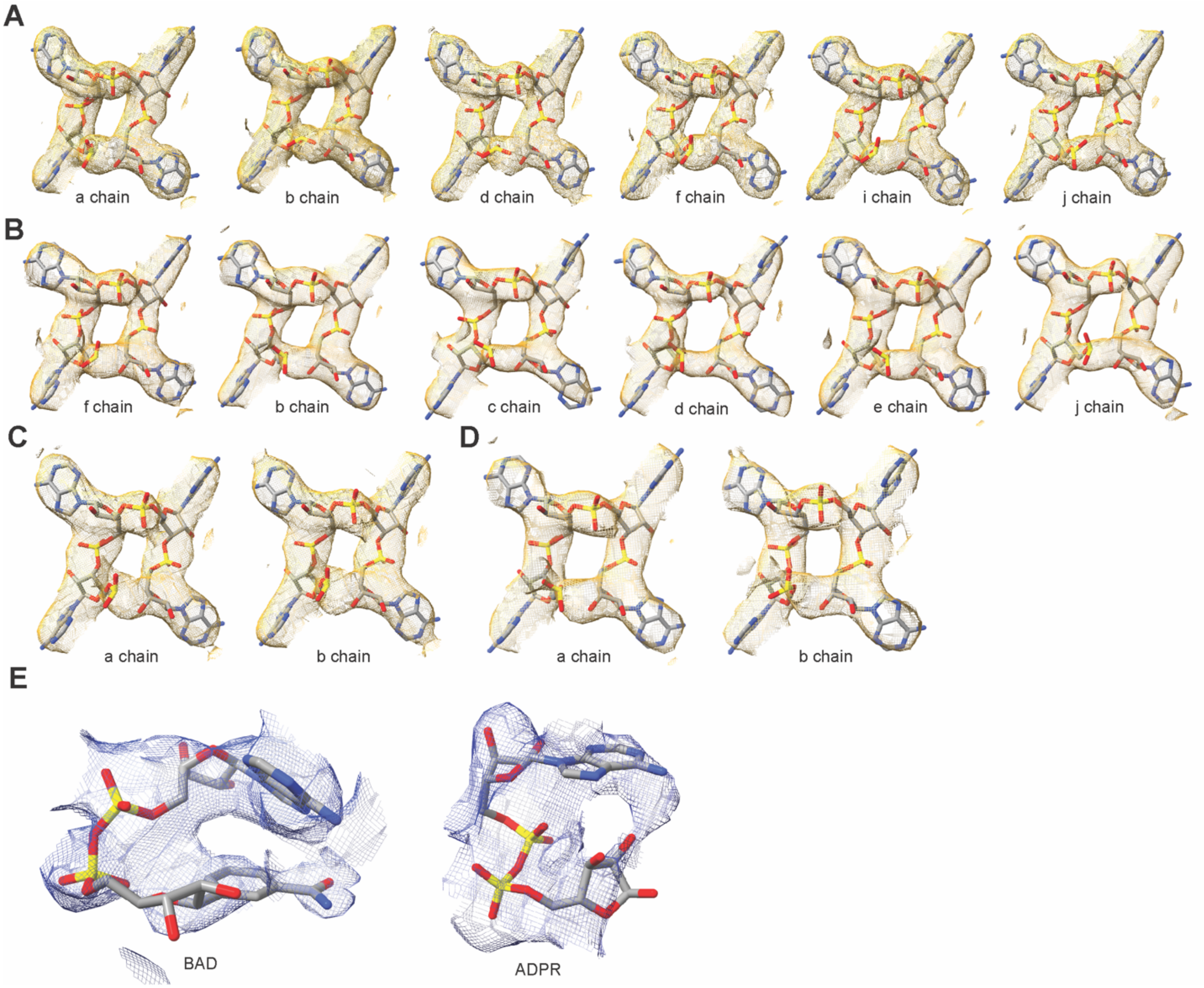

Fig. S9.

Coulomb potential maps of cA_4, BAD and ADPR. (A) Displays of six representatives of the cA_4 density from cA_4-Cat1 trigonal filament map in the mesh presentation (contour level 8.7σ). **(B)** Similar representation of six cA_4 density from cA_4-Cat1 pentagonal filament map (contour level 6.4σ). **(C)** cA_4 density from cA_4-Cat1-BAD map (contour level 6.3σ). **(D)** cA_4 density from cA4-Cat1-NAD map at (contour level 8.9σ). (E) BAD and ADPR density from cA_4-Cat1-BAD map (contour level 2.9σ) and cA4-Cat1-NAD map (contour level 4σ).

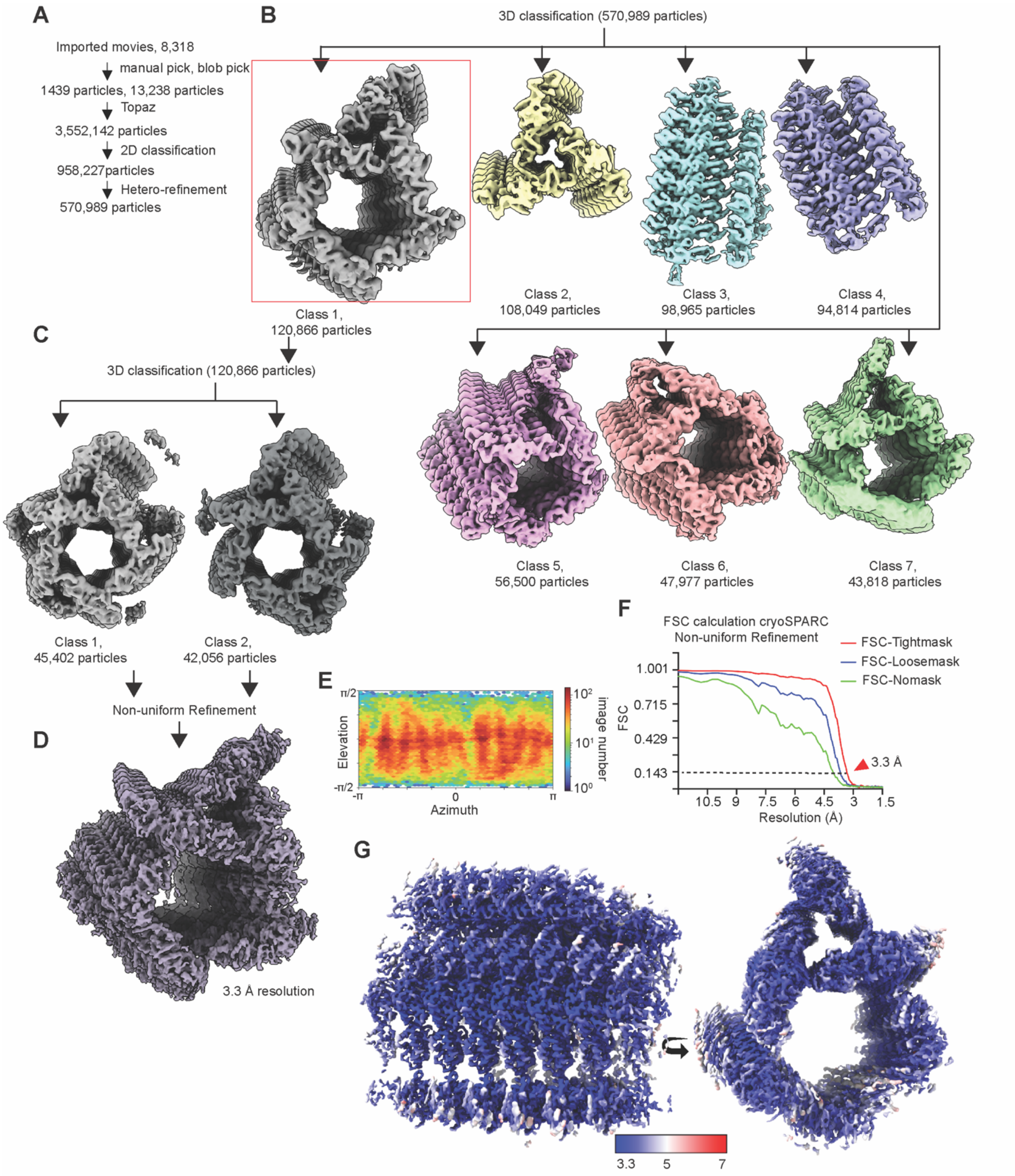

Fig. S10.

Cryo-EM data processing workflow of the cA4-Cat1-BAD structure determination. (A) cA_4-Cat1-BAD data processing workflow is represented with the number of particles mentioned for each step. **(B)** Seven 3D classes are displayed with particle number mentioned for each class. **(C)** The particles belong to class1 from **(B)** is further classified into two classes. **(D)** The particles from both classes (displayed in **C)** were merged and refined using non-uniform

refinement to 3.3 Å resolution. **(E)** Angular distribution of the particles used to obtain the final map. **(F)** The Fourier shell correlation (FSC) curves with the tight mask, loose mask and no mask estimated by non-uniform refinement job is plotted and the resolution at 0.143 FSC value is pointed by the red arrowhead. **(G)** Local resolution estimated by cryoSPARC local resolution estimation job is displayed. The scale bar is presented in Å unit.

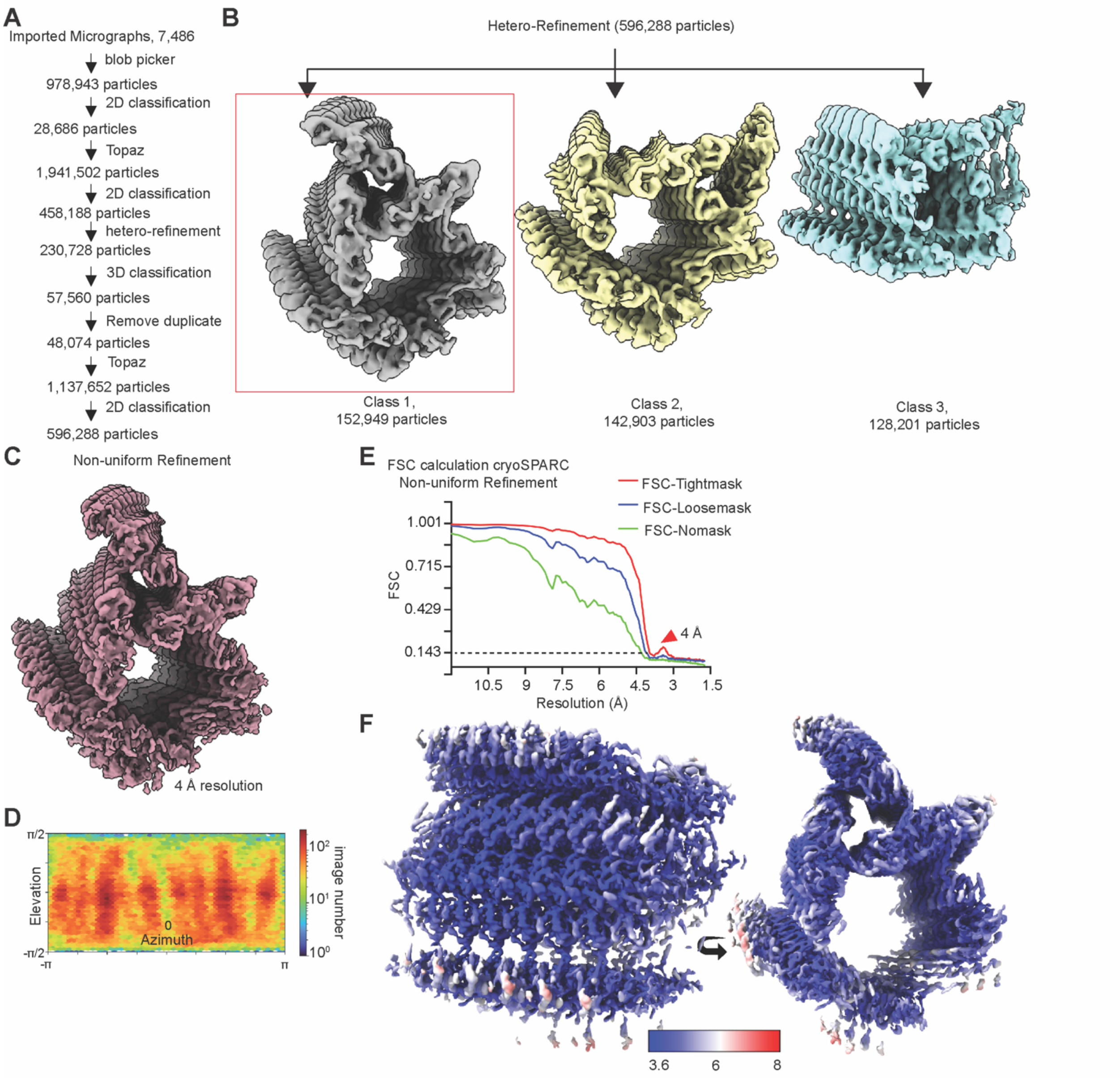

Fig. S11.

Cryo-EM data processing workflow of the cA4-Cat1-NAD$^+$ structure determination. (A) cA_4-Cat1-NAD data processing workflow is presented, and the number of particles selected by each step is mentioned. **(B)** The 3D maps were classified by hetero-refinement job and three of the maps are shown with the corresponding particle numbers. **(C)** Class 1 from **(B)** was selected for the non-uniform refinement job and refined to 4 Å resolution. **(D)** Angular distribution of the particles used to obtain the final map. **(E)** FSC curves calculated by the non-uniform refinement job are plotted for the tight mask, loose mask and no mask and the resolution at 0.143 FSC is indicated by the red arrowhead. **(F)** The local resolution estimation was performed using local resolution estimation job and the map is displayed. The scale bar is presented in Å unit.

	cA_4-Cat1 (Trigonal) (PDB 9MW9) (EMDB 48698)	cA_4-Cat1 (Pentagonal) (PDB 9MUD) (EMDB 48629)	cA_4-Cat1-BAD (Pentagonal) (PDB 9MUO) (EMDB 48639)	cA_4-Cat1-NAD (Pentagonal) (PDB 9MUE) (EMDB 48630)
**Data collection and Processing (for each dataset)**				
Microscope	Titan Krios	Titan Krios	Krios G4	Titan Krios
Voltage (keV)	300	300	300	300
Camera	Falcon IV	Falcon IV	Falcon 4i	Gatan K3
Magnification	165000	165000	165000	81000
Pixel size at detector (Å/pixel)	0.73	0.73	0.725	0.856
Total electron exposure ($e^{-}/Å^2$)	45	45	59.33	57.32
Exposure rate (e-/pixel/sec)	4	4	11.5	20
Number of frames collected during exposure	60	60	45	50
Defocus range (μm)	-0.8 to -2	-0.8 to -2	-0.8 to -2.3	-1 to -2.5
Phase plate (if used)	-	-	-	-
- phase shift range (in degrees)	-	-	-	-
- number of images per phase plate position	-	-	-	-
Automation software (EPU, SerialEM or manual)	Leginon (NCCAT)	Leginon (NCCAT)	EPU	Leginon (NCCAT)
Tilt angle (if grid was tilted)	-	-	-	-
Energy filter slit width (eV)	20	20	10	20
Micrographs collected (no.)	10,749	10,749	8,318	7,486
Micrographs used (no.)	10,749	10,749	8,318	7,486
Total extracted particles (no.)	3,068,364	3,068,364	3,552,142	1,941,502
**<u>For each reconstruction:</u>**				
Refined particles (no.)	84,493	12,721	87,458	152,750
Final particles (no.)	84,493	12,721	87,458	152,750
Point-group or helical symmetry parameters	-	-	-	-
Estimated error of translations/rotations (if available)	-	-	-	-
Resolution (global, Å)	3	3.4	3.3	4
FSC 0.5 (unmasked/masked)	-	-	-	-
FSC 0.143 (unmasked/masked)	3.8/3	4.7/3.4	4/3.3	4.3/4
Resolution range (local, Å)	3-7	3.4-9	3.3-7	3.6-8
Resolution range due to anisotropy (Å)	-	-	-	-
Map sharpening *B* factor ($Å^2$) / (B factor Range)	-	-	-	-
Map sharpening methods	-	-	-	-
**Model composition (for each model)**				
Protein	5478	7470	1012	1012
Ligands	0	0	1	2
RNA/DNA	44	60	8	8
**Model Refinement (for each model)**				
Refinement package	Phenix	Phenix	Phenix	Phenix
- real or reciprocal space	Real space	Real space	Real space	Real space
- resolution cutoff	3	3.4	3.3	4
Model-Map scores				
-CC	0.89 (mask)	0.86 (mask)	0.85 (mask)	0.86 (mask)
- Average FSC (d FSC at 0.143)	3.1	3.7	3.4	4
B factors ($Å^2$)				
Protein residues	167.64	173.70	130.28	120.32
Ligands	-	-	214.76	155.45
RNA/DNA	123.42	135.63	58.10	84.98

R.m.s. deviations from ideal values				
Bond lengths (Å)	0.004	0.003	0.004	0.004
Bond angles (°)	0.759	0.720	0.774	0.716
**Validation (for each model)**				
MolProbity score	2.1	1.98	2.02	2.12
CaBLAM outliers	2.07	2.34	3.04	4.25
Clashscore	12.84	15.77	13.49	16.02
Poor rotamers (%)	2.01	0.47	0.67	0.11
C-beta deviations	0	0	0	0
EMRinger score (if better than 4 Å resolution)	-	-	-	-
Ramachandran plot				
Favored (%)	96.25	95.96	94.40	93.80
Outliers (%)	0.00	0.00	0.20	0.10

Table S1.
Cryo-EM data collection, refinement, and validation statistics.

Data S1. (separate file)
Cat1 homologs and their association with type III CRISPR-Cas systems.
